# Action potential processing in a detailed Purkinje cell model reveals a critical role for axonal compartmentalization

**DOI:** 10.3389/fncel.2015.00047

**Published:** 2015-02-24

**Authors:** Stefano Masoli, Sergio Solinas, Egidio D'Angelo

**Affiliations:** ^1^Department of Brain and Behavioral Science, University of PaviaPavia, Italy; ^2^Brain Connectivity Center, Istituto Neurologico IRCCS C. MondinoPavia, Italy

**Keywords:** Purkinje cell, cerebellum, modeling, action potential, ionic channels

## Abstract

The Purkinje cell (PC) is among the most complex neurons in the brain and plays a critical role for cerebellar functioning. PCs operate as fast pacemakers modulated by synaptic inputs but can switch from simple spikes to complex bursts and, in some conditions, show bistability. In contrast to original works emphasizing dendritic Ca-dependent mechanisms, recent experiments have supported a primary role for axonal Na-dependent processing, which could effectively regulate spike generation and transmission to deep cerebellar nuclei (DCN). In order to account for the numerous ionic mechanisms involved (at present including Nav1.6, Cav2.1, Cav3.1, Cav3.2, Cav3.3, Kv1.1, Kv1.5, Kv3.3, Kv3.4, Kv4.3, KCa1.1, KCa2.2, KCa3.1, Kir2.x, HCN1), we have elaborated a multicompartmental model incorporating available knowledge on localization and gating of PC ionic channels. The axon, including initial segment (AIS) and Ranvier nodes (RNs), proved critical to obtain appropriate pacemaking and firing frequency modulation. Simple spikes initiated in the AIS and protracted discharges were stabilized in the soma through Na-dependent mechanisms, while somato-dendritic Ca channels contributed to sustain pacemaking and to generate complex bursting at high discharge regimes. Bistability occurred only following Na and Ca channel down-regulation. In addition, specific properties in RNs K currents were required to limit spike transmission frequency along the axon. The model showed how organized electroresponsive functions could emerge from the molecular complexity of PCs and showed that the axon is fundamental to complement ionic channel compartmentalization enabling action potential processing and transmission of specific spike patterns to DCN.

## Introduction

Neurons are the most complex cells, from a biochemical and biophysical point of view, of the entire human body. Neuronal functions critically depend on their ionic channels, expressed in different subtypes and selectively distributed in different cell sections, and on an intricate network of intracellular regulatory systems (Koch, 1999). A prototypical case of neuronal complexity is the Purkinje cell (PC) of the cerebellum. Discovered in the 19th century by Jan Evangelista Purkinje (Zárskı, 2012) this neuron was then described by Golgi and Cajal (De Carlos and Borrell, 2007) and deeply investigated in seminal works (Eccles et al., 1966; Llinas and Sugimori, 1980a). The electroresponsive mechanisms were outlined in the 80's and the first detailed model, attempting a biophysical reconstruction of PC functions based on realistic morphology (Rapp et al., 1994) and distributed ionic mechanisms, was presented in the 90's (De Schutter and Bower, 1994a,b). However, new critical functional features and molecular properties have been discovered since then and the biophysical mechanisms of PC electroresponsiveness need now to be reassessed.

Experimental recordings *in vivo* and *in vitro* have shown that, in PCs, multiple functional aspects coexist: the PCs (i) are autorhythmic *in vitro* (Raman and Bean, 1999; Khaliq et al., 2003) and *in vivo* (Shin et al., 2007), (ii) show an almost linear input-output relationship with current injection until they (iii) generate complex-bursting, and (iv) can move between *up* and *down* states in certain functional conditions (Loewenstein et al., 2005; Schonewille et al., 2006; Rokni et al., 2009). Moreover, (v) PC basal frequency discharge can be modulated by the expression of *Zebrin*-linked proteins (Z+ and Z- PCs; Zhou et al., 2014) and variant firing patterns have been observed, including “continuously firing” and “pausing” PCs (CF-PC and P-PC; Yartsev et al., 2009) as well as PCs showing very high-frequency bursts alternated to silent periods (Cheron et al., 2014). The PCs electroresponsive properties are the basis to explain synaptic integration, during which simple spikes, complex spikes, and *up* and *down* states occur in various combinations. Therefore, a new model of PC electroresponsiveness accounting for all these functional aspects is much needed.

The original hypothesis about the role of dendritic Ca channels in promoting PC firing (Llinas and Sugimori, 1980a) has been recently revisited by showing that Na channel distribution among axonal initial segment (AIS), soma and Ranvier nodes (RNs) is critical (Khaliq et al., 2003; Clark et al., 2005; Palmer et al., 2010). Moreover, the axon was shown to filter PC spike frequency limiting the effective communication with deep cerebellar nuclei (DCN) neurons, but it remained unclear whether this was due to Na channel inactivation or other membrane mechanisms (Monsivais et al., 2005; Yang and Wang, 2013). Another open issue concerns the role that numerous K channels might play in regulating PC firing (Martina et al., 2003; Khavandgar et al., 2005; McKay et al., 2005; Chang et al., 2007; Womack, 2010; Hosy et al., 2011). Finally, the conditions allowing bistability to emerge are still debated (Loewenstein et al., 2005; Schonewille et al., 2006; Rokni et al., 2009).

In the last two decades, more than 15 voltage-activated and second messenger-activated ionic channels have been identified in PCs. These include Nav1.6, Cav2.1, Cav3.1, Cav3.2, Cav3.3, Kv1.1, Kv1.5, Kv3.3, Kv3.4, Kv4.3, KCa1.1, KCa2.2, KCa3.1, Kir2.x, HCN1 (Khaliq et al., 2003; Swensen and Bean, 2003; Akemann and Knopfel, 2006; Angelo et al., 2007; Anwar et al., 2010). Several of these channels have been investigated through combined electrophysiological and pharmacological measurements and through selective mutations in mice, suggesting their role in determining the electrophysiological properties of PCs. This novel molecular complexity also requires to be integrated into a framework explaining action potential processing.

We have faced these questions by developing a realistic PC model based on the extensive biological information available. The PC model, once implemented with an accurate representation of axonal compartments, simultaneously reproduced autorhythmicity, simple spike frequency modulation and complex bursting. Axonal and somatic Na channels were critical for simple spike generation and sustained firing, dendritic Ca channels contributed to sustain pacemaking and complex bursting, axonal K channels were critical for spike frequency filtering. Bistability was incompatible with the remaining functions and emerged upon down-regulation of Na and Ca channels. The model thus provided a coherent hypothesis on how ionic channel localization and function regulates action potential generation and propagation, highlighting a crucial role for axonal compartmentalization.

## Methods

We have implemented an advanced multicompartmental PC model in Python-NEURON (Python 2.7; NEURON 7.3; Hines et al., 2007, 2009). Simulations were performed on eight cores AMD FX 8350 CPU (16 GB ram) and on a 72 cores/144 threads cluster (six blades with two Intel Xeon X5650 and 24 Gigabyte of DDR3 ram per blade). During simulations, the time step was fixed at 0.025 ms and the NEURON multi-split option was used to distribute computation corresponding to cell compartments over different cores (http://www.neuron.yale.edu/phpBB/) (Hines and Carnevale, 2008). With this arrangement it was possible to run up to six 15-s simulations in parallel in less than 40 min.

### Model construction

**Figure 1:**
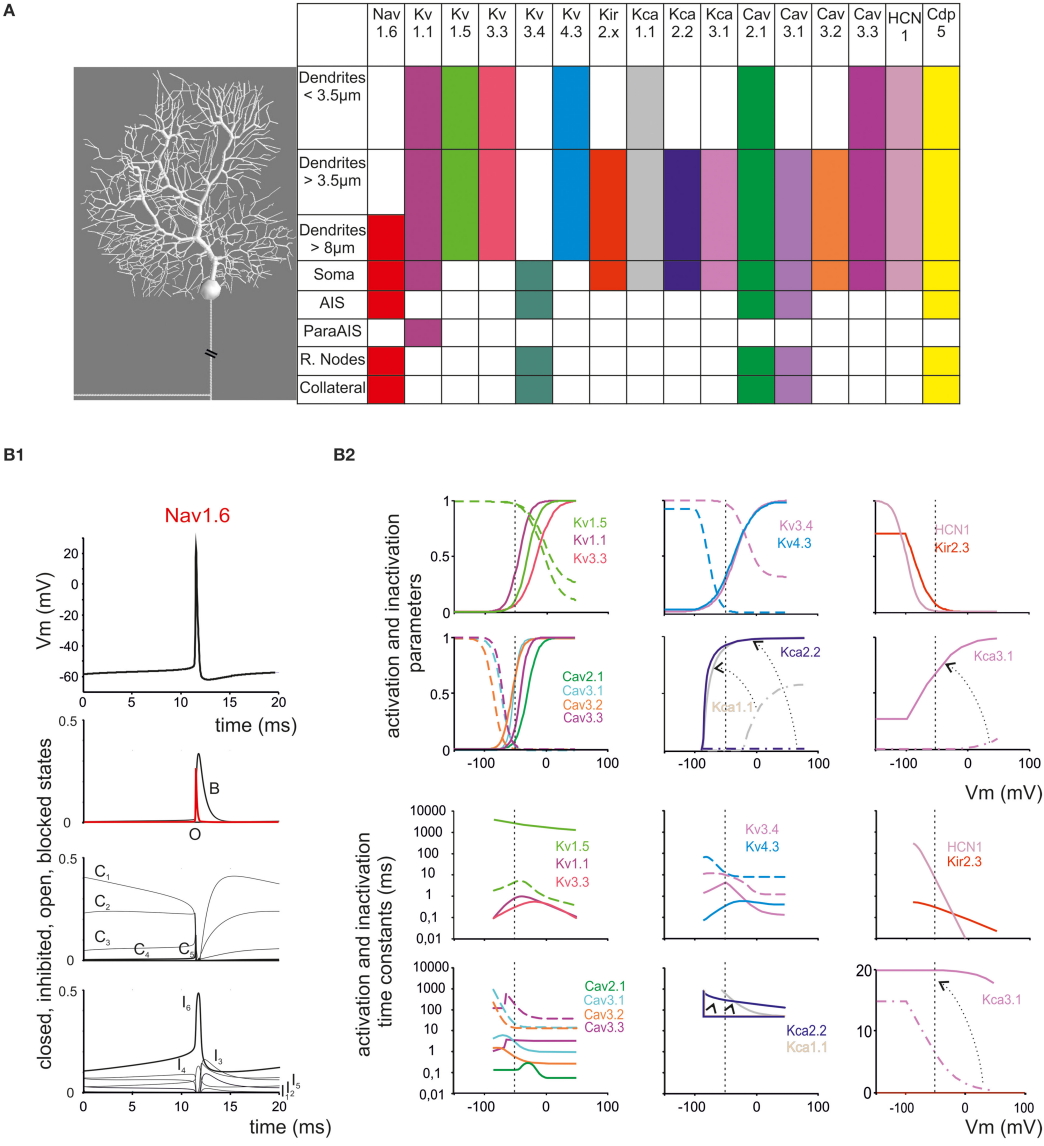
**Ionic channel types, distribution and gating properties in the PC model. (A)** *Left:* schematic drawing showing the morphology of the PC model reconstructed according to data reported in Table 1. *Right*: the PC model is divided into eight electrotonic sections and endowed with specific ionic mechanisms according to immunohistochemical data. Ionic channels include Na, K and Ca channels (Nav1.6, Cav2.1, Cav3.1, Cav3.2, Cav3.3, Kv3.4, Kv1.1, Kv4.3, Kv1.5, Kv3.3, KCa1.1, KCa3.1, KCa2.2, Kir2.x, HCN1) and a Ca buffering system (CDP5). **(B)** Ionic channel gating parameters are shown according to equations reported in **Tables 2**, **3**. **(B_1_)**: state variables of the Nav1.6 channels during an action potential. C, I, O, B, indicate closed, inactivated, open and blocked states. **(B_2_)** Steady-state activation and inactivation parameters (top) and activation and inactivation time constants (bottom) for Ca and K channels. For Kca3.1, Kca1.1, and KCa2.2, two gating curves are shown and arrows indicate gating occurring when changing Ca concentration (Kca1.1 and KCa2.2 from 0.045 μM to 1.6 μM Ca; KCa3.1 from 0.045 μM to 10 μM Ca; cf. **Figure 6**). Continuous lines indicate activation, broken lines indicate inactivation (when present). Same colors as in panel **(A)**. Vertical dashed lines indicate the approximate action potential threshold (−50 mV).

The model consisted of somatic, dendritic and axonal compartments generating a morpho-electrical equivalent of the PC (Figure 1A; Table 1). The voltage- and Ca^2+^-dependent mechanisms (Figure 1B; see below) were distributed among the compartments. With this approach, the model reproduced satisfactorily PC electroresponsiveness elicited by somatic current injection. It should be noted that the existence of PC variants was suggested based on histochemical and electrophysiological analysis (McKay and Turner, 2005; Kim et al., 2012, 2013). Here we have reconstructed a *canonical* PC model, which simulates the typical electrophysiological behavior of PCs.

**Table 1:** **Electrotonic compartments in the PC model**.

**Section name**	**Diameter (μm)**	**Length (μm)**	**N° sections**
Dendrites	0.67–9.22	1–10	1599
Soma	29.8	29.8	1
AIS	0.97	17	1
ParaAIS	0.97	4	1
Myelin	0.73	100	4
Nodes	0.73	4	3
Collateral	0.6	100	2

The table shows the sections of the PC model along with their number, diameter and length.

The ionic conductances were distributed over model compartments (Figures 1A,B; Tables 2, 3). In order to keep all gating kinetics at 37°C, kinetic constants reported in different papers were corrected from T_exp_ (experimental temperature) to T_sim_ (model temperature) using the equation Q^(Tsim—Texp)/10^_10_ and Q_10_ = 3 (Gutfreund and Segev, 1995). Nernst equilibrium potentials were pre-calculated from ionic concentrations used in current-clamp recordings and maintained fixed, except for the Ca^2+^ equilibrium potential, which was updated during simulations according to the Goldman-Hodgkin-Katz equation. The maximum ionic conductances were regulated to match the PC responses to various stimulations (Traub and Llinas, 1979; Traub and Michelson, 1991; Vanier and Bower, 1999; Achard and De Schutter, 2006; Druckmann et al., 2007, 2008, 2011, 2013; Solinas et al., 2007a,b). Ionic channel gating was modeled following the Hodgkin and Huxley formulation or Markov-chains for multi-state transitions using mathematical methods reported previously (D'Angelo et al., 2001; Nieus et al., 2006; Solinas et al., 2007a,b; Anwar et al., 2010) (see Table 1). In each compartment, membrane voltage was obtained as the time integral of the equation (Yamada, 1989):

(1) $$\frac{dV}{dt}=-\frac{1}{C_{m}}*\left\{\sum \left[g_{i}*\;\left(V-V_{i}\right)\right]+i_{inj}\right\}$$

**Table 2:** **Ionic mechanisms in the PC model: I**.

**Conductance/Location**		**Gmax (S/cm^2^)**	**Erev (mV)**	**Description of channel (H.H or Markovian)**	**References**
**Na CHANNEL**					
Nav1.6	Dendrites	0.016	60	Markovian 13 states	Raman and Bean, 2001
	Soma	0.214			
	AIS	0.5			
	Nodes	0.03			
	Collateral	0.03			
**K CHANNELS**					
Kv1.1	Dendrites	0.0012	−88	HH	Akemann and Knopfel, 2006
	Soma	0.002			
	ParaAIS	0.01			
Kv1.5	Dendrites	1.3*10^−4^	−88	HH	Courtemanche et al., 1998
Kv3.3	Dendrites	0.01	−88	HH	Akemann and Knopfel, 2006
Kv3.4	Soma	0.05	−88	HH	Raman and Bean, 2001; Khaliq et al., 2003
	AIS	0.01			
	Nodes	0.01			
	Collateral	0.02			
Kv4.3	Dendrites	0.001	−88	HH	Diwakar et al., 2009
Kir2.x	Dendrites	0.00001	−88	HH	Diwakar et al., 2009
	Soma	0.00003			
**Ca DEPENDENT K CHANNELS**					
Kca1.1	Dendrites	3.5*10^−2^	−88	Markovian	Anwar et al., 2010
	Soma	0.01			
Kca2.2	Dendrites	1*10^−3^	−88	Markovian	Solinas et al., 2007a,b
	Soma	1*10^−3^			
Kca3.1	Dendrites	0.002	−88	HH	Rubin and Cleland, 2006
	Soma	0.01			
**Ca CHANNELS**					
Cav2.1	Dendrites	1*10^−3^	137.5	HH	Swensen and Bean, 2005; Anwar et al., 2010
	Soma	2.2*10^−4^			
	AIS	2.2*10^−4^			
	Nodes	2.2*10^−4^			
	Collateral	2.2*10^−4^			
Cav3.1	Dendrites	5*10^−6^	137.5	HH	Anwar et al., 2010
	Soma	7*10^−6^			
	AIS	1*10^−5^			
	Nodes	1*10^−5^			
	Collateral	1*10^−5^			
Cav3.2	Dendrites	0.0012	137.5	HH	Huguenard and McCormick, 1992
	Soma	0.0008			
Cav3.3	Dendrites	0.0001	137.5	HH	Xu and Clancy, 2008
	Soma	0.0001			
**MIXED CATIONIC CHANNEL**					
HCN1	Dendrites	0.000004	−34.4	HH	Angelo et al., 2007; Larkum et al., 2009
	Soma	0.0004			
**CALCIUM BUFFER—PUMPS DENSITY**					
Ca Buffer	Dendrites	2*10^−8^		Markovian	Anwar et al., 2010
	Soma	5*10^−8^			
	AIS	5*10^−8^			
	Nodes	5*10^−7^			
	Collateral	5*10^−8^			

The table shows ionic channel localization, maximum conductance and reversal potential. Gating equations were written either in Hodgkin-Huxley (HH) style or Markovian style according to indicated references.

**Table 3:** **Ionic mechanisms in the PC model: II**.

**Conductance state variables**		**n**	**α (s^−**1**^)**	**β (s^−**1**^)**
Nav1.6	Open		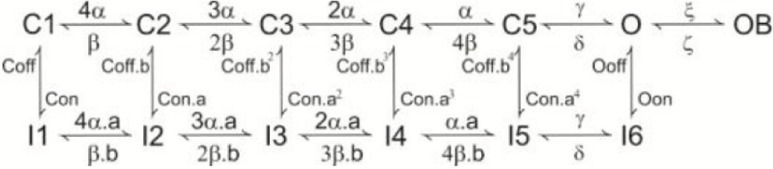	
	Blocked			
	Closed			
	Inactivated			
Kv1.1	Activation	4	$\text{Alphan}=0.12889\;*\;{\text{exp}}^{\frac{-(\text{v}+45)}{\;-33.90877}}$	$\text{Betan}=0.12889\;{*\text{exp}}^{\frac{-\left(\text{v}+45\right)}{12.42101}}$
			$\text{Ninf}=\frac{0.12889\;{*\text{exp}}^{\frac{-(\text{v}+45)}{\;-33.90877}}}{0.12889*{\text{exp}}^{\frac{-(\text{v}+45)}{\;-33.90877}}\;+\;0.12889\;{*\text{exp}}^{\frac{-(\text{v}+45)}{12.42101}}}$	$\text{Taun}=\frac{1}{(\text{qt})\;*(0.12889)\;*{\text{exp}}^{\frac{-(\text{v}+45)}{\;-33.90877}}+\;0.12889\;{*\text{exp}}^{\frac{-(\text{v}+45)}{12.42101}}}$
Kv1.5	Activation	3	$\text{Malp}=\frac{\text{q}10\;*0.65}{{\text{exp}}^{\frac{-(\text{v }+\;10)}{8.5}}+{\text{exp}}^{\frac{-(\text{v }-\;30)}{59}}}$	$\text{Mbet}=\frac{\text{q}10\;*0.65}{2.5\;{+\text{exp}}^{\frac{\text{v }+\;82}{17}}}$
	Inactivation	1	$\text{Nalp}=\frac{0.001\;*\text{q}10}{2.4\;+10.9\;{*\text{exp}}^{\frac{-\text{v }+\;90}{78}}}$	$\text{Nbet}=\text{q}10\;*0.001\;{*\text{exp}}^{\frac{\text{v }-\;168}{16}}$
				$\text{Nce}=1\;*\frac{0.25+1}{1.35\;{+\text{exp}}^{\frac{\text{v }+\;7}{14}}}$
	Inactivation	1	$\text{Mce}=\frac{1}{1+{\text{exp}}^{\frac{-\text{v }+\;30.3}{9.6}}}$	$\text{Uce}=1\;*\frac{0.1+1}{1.1\;{+\text{exp}}^{\frac{\text{v }+\;7}{14}}}$
			$\text{Mtau}=\frac{1}{\frac{\text{a }+\text{ b}}{3}}\;*\text{Tauact}$	$\text{Ntau}=\frac{1}{\frac{\text{a }+\text{ b}}{3}}\;*\text{Tauinactf}$
				Uau=6800 *Tauinacts
Kv3.3	Activation	4	$\text{Alpha}=0.22\;{*\text{exp}}^{\frac{-(\text{v}+16)}{-26.5}}$	$\text{Beta}=0.22\;{*\text{exp}}^{\frac{-(\text{v}+16)}{26.5}}$
				$\text{Taun}=\frac{1}{0.22\;{*\text{exp}}^{\frac{-(\text{v}+16)}{-26.5}}+0.22\;{*\text{exp}}^{\frac{-(\text{v}+16)}{26.5}}}$
Kv3.4	Activation	3	$\mathrm{Minf}=\frac{1}{1+exp^{\frac{-v+24}{15.4}}}$	$\mathrm{Hinf}=0.\;31\;+\;\frac{0.69}{1+exp^{\frac{v-\;(-5.802)}{11.2}}}$
	Inactivation	1	$\begin{array}{l} \text{Mtau}=1000\;*\;\frac{mtau\_func(v)}{qt}\\ \;If\;Vm<-35 \end{array}$	$\begin{array}{l} Htau=\frac{1000\;*\;htau\_func(v)}{qt}\\ \;If\;Vm>0 \end{array}$
			$\begin{array}{l} Mtau=(3.4225e^{-5}+0.00498exp^{\frac{-v}{-28.29}})\;*3\\ \;else\\ Mtau=0.00012851+\frac{1}{exp^{\frac{v+\;100.7}{12.9}}+exp^{\frac{v+\;-56.0}{-23.1}}} \end{array}$	$\begin{array}{l} Htau=0.0012+0.0023\;*exp^{-0.141*Vm}\\ \;else\\ Htau=1.2202e^{-05}+\;0.012\;*\;exp^{\frac{-(Vm-(-56.3))}{49.6^{2}}} \end{array}$
Kv4.3	Activation	2	Alp_a=Q10∗0.8147∗sigm(v-(-9.17203,−23.32708))	$Bet\_a=\frac{Q10*\;0.1655}{exp^{\frac{v-(\;-18.27914)}{19.47175}}}$
	Inactivation	1	$\begin{array}{l} \text{Alp}\_\text{b}=\text{Q}10*0.0368*\text{sigm}(\text{v}-(-111.33209,12.8433))\\ Tau\_a=\\ \frac{1}{\begin{array}{c} q10*0.8147*sigm\left(v-\;\left(-9.17203,\;-23.32708\right)\right)+\;\\ Q10*\;\frac{0.1655}{exp\frac{v-(-18.27914)}{19.47175}} \end{array}} \end{array}$	$\begin{array}{l} Bet\text{\_}b=Q\mathrm{10}*\mathrm{0.0345}*sigm(v-(-\mathrm{49.9537},-\mathrm{8.90123}))\\ Tau\_b=\\ \frac{1}{\begin{array}{c} Q10*\;0.0368\;*sigm(v-\;(-111.33209,\;12.8433))+\;\\ Q10*\;0.0345*sigm(v-\;(-49.9537,\;-8.90123))\; \end{array}} \end{array}$
Kir2.x	Activation	1	$Alp\_d=Q10*\;0.13289*exp^{\frac{v-\;(-83.94)}{-24.3902}}$	$\begin{array}{l} \;Bet\text{\_}d=Q10*0.\mathrm{16994}*exp^{\frac{v-\;(-83.94\;)}{35.714}}\\ Tau\_d=\\ \frac{1}{Q10*\;0.13289*exp^{\frac{v-\;(-83.94)}{-24.3902}}+\;Q10*\;0.16994*exp^{\frac{v-\;(-83.94\;)}{35.714}}} \end{array}$
Kca1.1	Open/closed		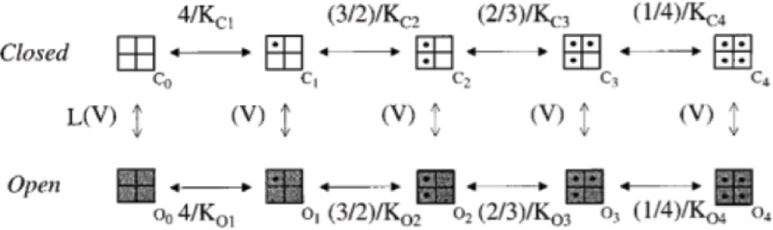	
Kca2.2	Open/closed		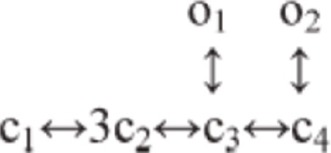	
Kca3.1			$\begin{array}{l} \;Cai<\mathrm{0.01}\\ Yconcdep=500\left(\frac{1}{ms}\right)*\frac{0.015-cai*\left(\frac{1}{mM}\right)}{exp^{\frac{0.015-cai*1\left(\frac{1}{mM}\right)}{0.0013}}\;-1}\\ \;Yconcdep=500\left(\frac{1}{ms}\right)*\frac{0.005}{exp^{\frac{0.005}{0.0013}}\;-1}\\ \;TauY=\frac{1}{Yalpha\;+\;Ybeta} \end{array}$	
Cav2.1	Activation	3	$\begin{array}{l} \;If\;x>=-40\\ Taumfkt=0.2702+1.\mathrm{1622}*exp^{\frac{-(v+26.798)*(v+26.798)}{164.19}}\\ \;Taumfkt=0.\mathrm{6923}*exp^{\frac{v}{1089.372}} \end{array}$	
Cav3.1	Activation	2	Minf= 1/(1+exp((v+52)/−5))	Hinf=1/(1+exp((v+72)/7))
	Inactivation	1	$\tau_{m}=1+\frac{1}{\frac{exp\left(v+40\right)}{9}}+exp((v+102)/-18)$	$\tau_{h}=(15+1/exp((v+32)/7))$
Cav3.2	Activation	2	$M\_inf=\frac{1.0}{1\;+\;exp^{\frac{-(v+shift+54.8)}{7.4}}}$	$H\_inf=\frac{1.0}{1\;+\;exp^{\frac{v+shift+85.5}{7.18}}}$
	Inactivation	1	$Tau\_m=1.9\;+\;\frac{1.0}{exp^{\frac{v+shift+37.0}{11.9}}+\;\frac{exp^{\frac{-\left(v+shift+131.6\right)}{21}}}{5^{\frac{12}{10}}}}$	$Tau\_h=13.7\;+\;\frac{1942\;+\;exp^{\frac{v+shift+164}{9.2}}}{\frac{1\;+\;exp^{\frac{v+shift+89.3}{3.7}}}{3^{\frac{12}{10}}}}$
Cav3.3	Activation	2	$N\_inf=\frac{1}{{\left(1+exp\right)}^{\frac{-\left(v-vhalfn\right)}{kn}}}$	If v>−60
	Inactivation	1	$L\_inf\;=\frac{1}{1+exp^{\frac{-(v-vhalfl)}{kl}}}$	$\begin{array}{l} Tau\_n=\frac{7.2+0.02*exp^{\frac{-v}{14.7}}}{qt}\\ Tau\_l=\frac{79.5+2.0*exp^{\frac{-v}{9.3}}}{qt}\\ Tau\_n=\frac{0.875*exp^{\frac{v+120}{41}}}{qt}\\ \;Tau\_l=\frac{260}{qt} \end{array}$
HCN1	Activation	1	$h\;=\;\frac{1}{(0.0018)*\;(\;exp^{v-(\;-58,7)\;/\;-22})+\;exp\;\frac{v-(\;-58,7)}{7.14}\;/qt}$	

*The table reports the equations used to calculate membrane conductances used in the model. In most cases, the rate constants a_x_, b_x_, a_y_, and b_y_ were used to calculate x_*8*_, y_*8*_, τ_x_ and τ_y_ according to a Hodgkin-Huxley scheme (Hodgkin and Huxley, 1952). For each ionic channel i, the conductance g_i_ is*
$$g_{i}=Gmax_{i}*x_{i}^{zi}*y_{i}$$
*where, Gmax_i_ is the maximum ionic conductance, x_i_ and y_i_ are state variables (probabilities ranging from 0 to 1) for a gating particle, and z_i_ is the number of such gating particles. x and y (the suffix i omitted) were related to the first order rate constants a and ß by the equations*
$$x_{\infty }=\frac{\alpha_{x}}{alpha_{x}+\beta_{x}},y_{\infty }=\frac{\alpha_{y}}{\alpha_{y}+\beta_{y}};\tau_{x}=\frac{1}{\left(\alpha_{x}+\beta_{x}\right)},\tau_{y}=\frac{1}{\left(\alpha_{x}+\beta_{x}\right)}$$
*where a and b are functions of voltage.*

*The state variable kinetics were:*
$$\frac{dx}{dt}=\;\frac{\left(x_{\infty }-x\right)}{\tau_{x}},\frac{dy}{dt}=\frac{\left(y_{\infty }-y\right)}{\tau_{y}}$$

In some cases, the equations for x_∞_, y_∞_, τ_x_ or τ_y_ are reported directly. Exceptions to this general scheme were Markovian channel models for Nav1.6 and KCa1.1. Eventually, the probability of channel opening was transformed into ionic conductances and used to calculate the model currents. More details on the construction of this table were reported previously (D'Angelo et al., 2001; Solinas et al., 2007a; Subramaniyam et al., 2014).

Where *V* is membrane potential, *C_m_* membrane capacitance, *g_i_* are ionic conductances and *V_i_* reversal potentials (the subscript *i* indicates different channels), and *i_inj_* is the injected current. Adjacent compartments communicated through an internal coupling resistance (Diwakar et al., 2009).

#### Model morphology

***General PC structure.*** The model morphology was derived from a reconstruction of PC soma and dendrites obtained from a 2–3 months old Guinea pig (Rapp et al., 1994) used for a previous PC model (De Schutter and Bower, 1994a,b). This morphology was extended with the inclusion of the axon initial segment (AIS) and ParaAIS, of three nodes of Ranvier (RN) separated by four myelinated sections and of a 200 μm long axonal branch stemming from the second RN (Gianola et al., 2003; McKay and Turner, 2005; Foust et al., 2010; Palmer et al., 2010; Yang and Wang, 2013).

***Axonal compartments.*** The AIS is a portion of the axon laying between the soma and the first myelinated internode. Its length in PCs can vary between 15 and 20 micrometers (Somogyi and Hámori, 1976; Clark et al., 2005) and contains a high concentration of Na channels. The length of the AIS was set at 17 μm and the maximum Na channels density was carefully adapted to experimental values reported in neurons (Lorincz and Nusser, 2008; Foust et al., 2010; Zonta et al., 2011; Bender and Trussell, 2012; Bender et al., 2012). The paraAIS was modeled as a 4 μm portion of the axon adjacent to AIS and deprived of Na channels but enriched with K channels. The PC axon has a diameter of 0.5–1 μm, i.e., just enough to allow myelin to improve conductance speed (Debanne et al., 2011). The axon was implemented through four myelinated 100 μm compartments with 0.73 μm diameter, without any channels, and three RNs of 4 μm with 0.73 μm diameter endowed with a mixture of sodium and potassium channels (juxtaparanodes surrounding the RNs were not represented). During late morphogenesis the main axon generates a non-myelinated collateral 150–200 μm away from the soma (Clark et al., 2005; Sotelo and Rossi, 2013) which, for a short period during development, connects PCs on the horizontal plane (Maex and Steuber, 2013). This collateral has been also observed in adults healthy humans and proved five times more abundant in cases of “essential tremor” (Babij et al., 2013). Our model included a non-myelinated axonal collateral made of 2 identical 100 μm compartments with 0.6 μm diameter originating from the second RN.

#### Passive and active properties

The passive properties of the PC model can be summarized in three main values measurable in experiments: total PC area = 70,000 μm^2^, input resistance from soma *R*_in_ = 14*M*Ω input capacitance from soma *C*_*in*_ = 1090 pF. These properties are the result of the following parameter set: the specific capacitance (Cm) and the leakage for each section have been taken from the (De Schutter and Bower, 1994a,b) model. The Cm of all compartmentes was rescaled by 0.46 in order to achieve a specific membrane capacitance of the somatic compartment equal to 0.77 μF/cm^2^. The leakage was halved. The axial resistance has been set at 122 Ωcm for the entire model. This configuration gave an appropriate response to parallel fiber stimulation (not shown).

The PC model was endowed with 15 different types of voltage- and calcium-dependent ionic channels, which were differentially distributed in the cell compartments (Table 2). The corresponding membrane mechanisms were taken from published models and used without modifications of their kinetics (except for Q_10_ correction, see above; see Table 3 for mathematical representation). The maximum conductance of ionic channels was pre-adapted to experimental values and then tuned according to a procedure explained below. All the membrane mechanisms used in the model and in this paper have been named according to NC-IUPHAR (International Union of Basic and Clinical Pharmacology Committee on Receptor Nomenclature and Drug Classification; (Yu et al., 2005):

- Nav1.6—Voltage-dependent Na channel (including transient, persistent and resurgent components)
- Kv1.1, Kv1.5, Kv3.3, Kv3.4, Kv4.3—Voltage-dependent K channels (including delayed rectifier, A-type, M-type)
- Kca1.1, Kca 2.2 and Kca 3.1—Big, small and middle conductance calcium-dependent K channel
- Kir2.x—Inward rectifier K channel
- HCN1—Hyperpolarization activated cyclic nucleotide-gated cationic channel
- Cav3.1, Cav 3.2, Cav 3.3—Low voltage-activated (LVA) Ca channels (T-type)
- Cav2.1—High voltage-activated (HVA) Ca channel (P -type)
- *I_leak_*,—background leakage channel

#### Ionic mechanisms in the PC model

Since the whole PC *channellome* is not available yet, ionic channel descriptions have been partly obtained from PC recordings and partly from other neurons or expression systems.

***Nav1.6.*** We have adopted the Khaliq and Raman Nav1.6 sodium channel model including 13 kinetic states divided into four categories (closed, open, inactivated and blocked). As a specific property of Nav1.6, this model generates both the transient, persistent and resurgent current components (Khaliq et al., 2003). Instead of using the original version, which was lacking the Q10 parameters for some states transitions, we have implemented a modified version (Akemann and Knopfel, 2006). This allowed to tune the Na current to the actual model temperature (37°) preventing artifacts in spike shape. As reported in Table 1, Nav1.6 channels were placed in AIS, RNs, soma and main dendritic trunk.

***Kv1.1, 1.5, 3.3, 3.4, 4.3.*** The model of Kv1.1 is based upon the human gene (Akemann and Knopfel, 2006) and has been chosen because the kinetics resemble those from an eteromultimer Kv1.1 with different Kv1.2 subunits attached to it (Brew et al., 2007). It is present in the dendrites, soma and ParaAIS. The Kv1.5 model was taken from the Cardiac Atrial Cell model (the only suitable to our knowledge; (Courtemanche et al., 1998) with the NEURON implementation provided by Ingemar Jacobson (http://senselab.med.yale.edu/ModelDB/). The Kv3.3 model was taken from (Akemann and Knopfel, 2006) and was based on PC data (Martina et al., 2007) and lacks intrinsic prolonged inactivation. The Kv3.4 model was taken from the TEA-sensitive Purkinje potassium current (Khaliq et al., 2003) and was based on recordings from PCs (Raman and Bean, 1999). The Kv3.4 channel shows the same rapid activation/deactivation typical of the other Kv3 channels and shows fast inactivation. The phosphorylation normally operated by PKC is missing in the current model. Kv3.4 proved fundamental to control spike rate as well as the responses to high current injection and bursts. Kv3.3 was placed only in the dendrites, while Kv3.4 was placed in the same location as the sodium channel (Khaliq et al., 2003). The Kv4.3 channel (A-type) was taken from that elaborated for cerebellar granule cells (Diwakar et al., 2009) and has been placed in the dendritic tree.

***Kca1.1, 2.2 and 3.1.*** Kca1.1 (BK) was represented using a Markovian multi-state model derived from PC data (Anwar et al., 2010) and was placed in the dendrites and soma. Kca2.2 (SK2) was represented using a Markovian multi-state model derived from the Golgi cell (Solinas et al., 2007a,b) and was placed in part of the dendritic tree and in the soma. Kca3.1 (SK4) was taken from olfactory bulb mitral cells (Rubin and Cleland, 2006) and was placed in part of the dendritic tree and in the soma.

***Kir2.x.*** A Kir2.x model was taken from the cerebellar granule cell (Diwakar et al., 2009) approximating Kir2.3 expressed by PCs.

***Cav2.1.*** The Cav2.1 model was modified from (Anwar et al., 2010) and was based on PC data (Swensen and Bean, 2005). Cav2.1 was distributed in the entire model. It should be noted that Vhalf and K (−29.5 and −8.5 mV) are not identical to those reported by DeSchutter (Anwar et al., 2010). This accounts for the fact that original data were obtained at 22–24° rather than 37°. Moreover, other PCs recordings reported −34 and 4.8 mV (Watase et al., 2008). This HVA calcium channel has different splicing isoforms and can be modulated by caBP1 and Calmodulin, giving rise to CDF (calcium dependent facilitation) and CDI (calcium dependent inactivation) (Chaudhuri et al., 2007; Minor and Findeisen, 2010; Raghuram et al., 2012). The known subunits composition of PC Cav2.1 channels shows a Vhalf range between −27 and −30 with a K between 8 and 10 (He et al., 2007; Watase et al., 2008), as in our model (see Table 3).

***Cav3.1, 3.2, 3.3.*** The low voltage calcium channels were taken from different sources. Cav3.1 was taken from a previous PC model (Anwar et al., 2010) and used in a part of the dendritic tree, on the soma, AIS and RNs. Cav3.2 was taken from thalamic relay neurons (Huguenard and McCormick, 1992) and used on a part of the dendritic tree and soma. Cav3.3 was taken from CA3 pyramidal neurons (Xu and Clancy, 2008) and placed in the entire dendritic tree and soma.

***HCN1.*** The Hyperpolarization-activated cyclic nucleotide-gated channel HCN1 was taken from layer 5 pyramidal neurons (Angelo et al., 2007; Larkum et al., 2009) and used in the dendrites and soma.

***Calcium dynamics.*** Calcium dynamics were specifically coded for PCs by (Anwar et al., 2010) taking into account the passive diffusible calcium buffer Calbindin and Parvalbumin but not the active binding protein Calmodulin. The calcium pumps were modeled in a generic way (*The NEURON book*: http://ebooks.cambridge.org/ebook.jsf?bid=CBO9780511541612). Both buffer and pumps were placed along the entire model. The calcium buffer mechanism was designed to automatically adapt the number of shell inside the cytoplasm to the size of the corresponding model section.

***TRPC3.*** In some simulations, a TRP channel model taken from UBC neurons (Subramaniyam et al., 2014) was placed in the dendrites to represent TRPC3 channels (Zhou et al., 2014). The TRP channels was a leakage conductance with 0-mV reversal potential.

### Model tuning, testing, and validation

In the PC model, after defining the morphological and passive properties and setting the ionic channel complement with its subcellular and gating properties (*construction*), the free parameters remained the maximum ionic conductances of voltage- and calcium-dependent channels. These were pre-set based on experimental estimates taken from literature and fine-tuned through a trial-and-error approach (*tuning*). The matching of model output to experimental data was evaluated (*testing*) by comparing the voltage traces elicited in response to various sets of step-current injections including pulses from different holding potentials and responses to hyperpolarization. Parameter tuning was performed by varying the maximum conductances in turn. It will be of interest to evaluate how routines based on genetic algorithms (Druckmann et al., 2007, 2008, 2011, 2013) will solve the complex optimization problem of the PC model.

#### PC model tuning

A relevant issue in complex neuron models is to perform a precise calibration of the large number of ionic channel conductances involved. For example, in PCs different parameter combinations were shown to generate similar firing frequencies (Achard and De Schutter, 2006). In our reconstruction we have followed a procedure limiting the range of possible parameter combinations. The calibration of each conductance was guided by experimental indications and followed by specific tests determining the conditions for the existence of three fundamental functional properties: pacemaking, F/I relationship, complex bursting. Then, a robustness analysis was performed in order to estimate the confidence intervals of the maximum and minimum ionic conductances (Solinas et al., 2007a,b). In PCs, subtypes of Na, Ca and K channels provided strong constraints for model tuning, since their location, gating scheme and maximum conductance were often known from experimental measurements (Swensen and Bean, 2003). Therefore, the insertion of active properties proceeded in 4 steps, which were assisted by automatic *testing protocols* (see below).

- After setting morphology and passive properties, H channel maximum conductance was preset according to experimental measurements (Angelo et al., 2007) and its effect was evaluated on the hyperpolarizing responses.
- Na channels were located in the AIS, RN, soma and dendritic trunk in definite proportions. Then different types of K channels were added respecting the relative conductance and localization determined experimentally (Swensen and Bean, 2003). This gave the PC model the basic Na spike generating mechanism.
- The different Ca channels, the calcium buffering system and the different KCa channels were inserted (Anwar et al., 2010).
- Fine tuning was then performed to match the main electroresponsive properties of the model with experimental data.

#### Testing protocols

To test the model we developed two experimentally-based protocols aimed at reproducing the main PC properties in turn. Both protocols were run after each conductance change.

1. The first protocol was designed to test the ability of PCs to generate spontaneous firing and evaluated the PC model discharge in the absence of current injection. The AIS of PCs is the key section of the model, where the spikes are generate by a high concentration of sodium channels (Nav1.6). These channels, in strict conjunction with those in the soma, have the ability to push the cell over threshold generating spontaneous firing without current injections or synaptic activity (Foust et al., 2010; Palmer et al., 2010). If these channels are not properly set, the cell cannot generate spontaneous firing (Zonta et al., 2011) but is still able to generate spikes in response to current injections. If the PC model generated spontaneous firing without channels in the AIS, than the model was incorrect. The potential intervention of dendritic calcium entry as a cause of spontaneous firing (Llinas and Sugimori, 1980a, b) was tested by disconnecting the soma from the dendritic tree (Khaliq et al., 2003). The maximum spontaneous frequency allowed was 75 Hz (Walter et al., 2006). Subsequent protocols we valid only if this test was passed.
2. The second protocol was designed to evaluate the PC model electroresponsiveness upon somatic current injection. As in dissociated PCs (Khaliq et al., 2003), the model had to respond to a series of steps of current injection by increasing the spikes frequency generating a quasi-linear frequency/intensity (F/I) relationship up to about 300 Hz. At higher injected current intensities the PC model had to switch to “complex bursting” (Kim et al., 2012). Strong current injection in the soma (typically = 2.2 nA) should generates a calcium spike in the dendrites associated with sodium spike bursting. The duration, shape and amplitude of complex bursts were strictly dependent on dendritic Ca, K currents, and Kca currents.

#### Model robustness

The robustness of the model was measured by assessing its ability to maintain the typical PC electroresponsiveness when one out of the key parameters was varied (Solinas et al., 2007a,b) namely, spontaneous frequency (range 0–95 Hz), F/I relationship (±20 Hz compared to Khaliq et al., 2003), complex bursting (persistence beyond 2 s, 6 ± 1 burst/s, burst shape showing 4 ± 1 fast spikes after the Ca spike followed by a decreasing spike size with frequency of 250–300 Hz; cf. (McKay and Turner, 2005; Kim et al., 2012). This procedure was repeated for each ionic mechanism by systematically varying its maximum conductance. The evaluation of model robustness allowed to determine the criticality of ionic channels for the spike generation mechanism.

#### Data analysis

The PC model response properties were analyzed using MATLAB routines (MathWorks, Natick, MA, USA) based on data recorded during simulations. The area of the model has been calculated as the sum of each section area obtained with the NEURON built-in “area” function. The input resistance has been calculated using NEURON built-in “impedance” function.

## Results

A detailed model of PC electroresponsiveness was constructed and tested on the basis of morphological, biochemical, immunohistochemical and electrophysiological data, which allowed to preset the distribution and gating properties of ionic channels and then to fine tune maximum ionic conductances (see Methods). The model consisted of a somatic compartment, of 1599 dendritic compartments divided into three orders of branches, and of an axon made by AIS, paraAIS, three RNs, four myelinated internodes and a two-compartment axonal collateral (Figure 1A; Table 1). These compartments were endowed with 15 different types of voltage-and calcium-dependent ionic channels and a calcium buffering system (Figure 1B; Tables 2, 3). The PC structure was derived from a previous reconstruction (Rapp et al., 1994) and accounted for a total area of 70,000 μm^2^, an input resistance *R*_in_ = 14*M*Ω and an input capacitance of 1090 pF measured from soma.

### Electroresponsiveness of the PC model

**Figure 2:**
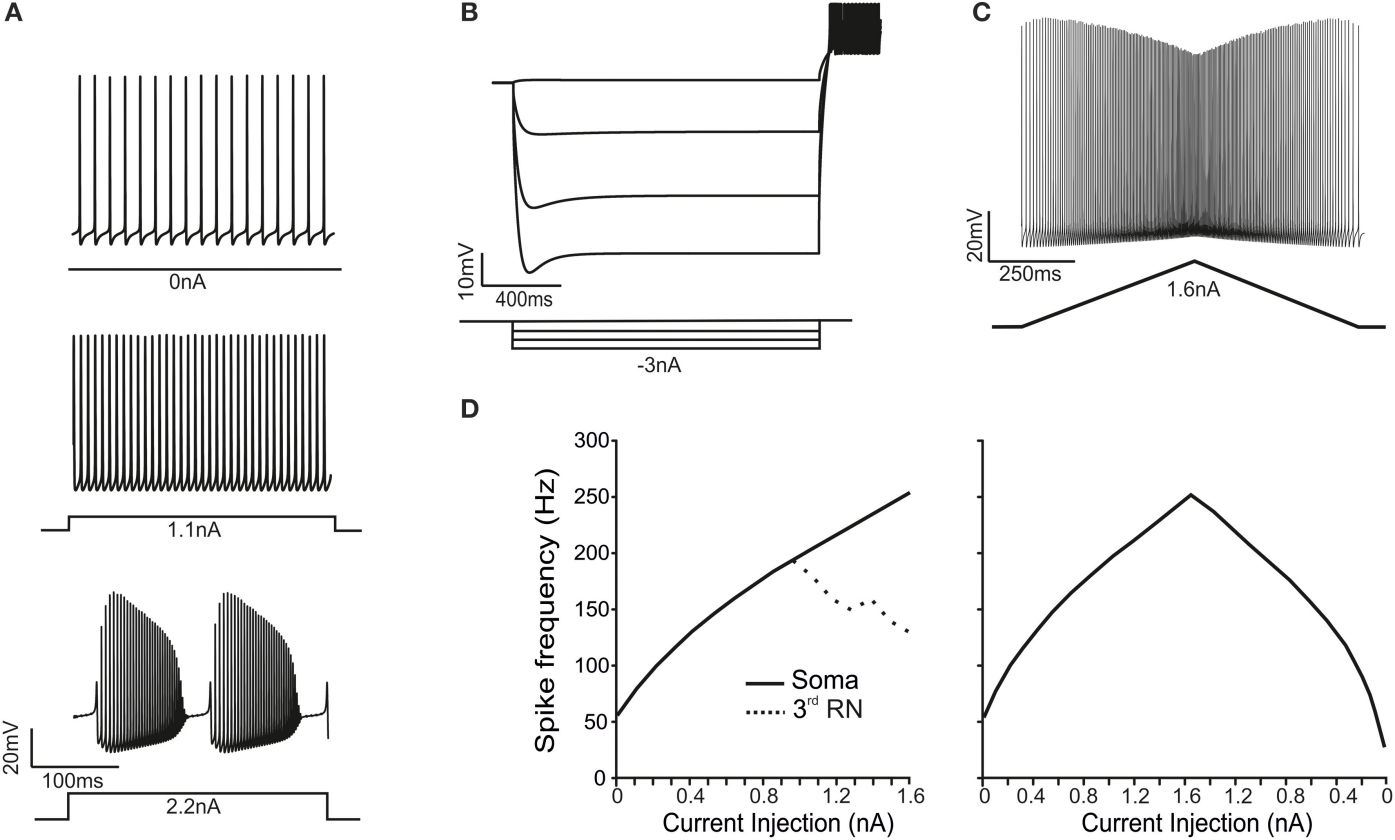
**Electroresponsive properties of the PC model. (A)** The traces show spikes in the soma during spontaneous firing and in response to moderate (1.1 nA) and high (2.2 nA) step-current injections in the soma, demonstrating the transition from simple spikes to complex bursting. **(B)** A series of negative step-current injections in the soma determines voltage responses showing the typical sag and rebound depolarization generated by the H-current. **(C)** A ramp-current injection (from 0 to 1.6 nA and back) causes a frequency-modulated response in the PC model. **(D)** In response to *step-current* injection from 0 to 1.6 nA (0.1 nA steps), the PC model generates proportionately higher spike frequencies. Conversely, the RNs are unable to sustain firing frequencies above 200 Hz (dotted line). In response to *ramp-current* injection increasing from 0 to 1.6 nA, the f-I curve closely resembles that obtained using step-currents. However, on the way back, the f-I curve is asymmetrical.

As observed in PCs *in vitro* (Raman and Bean, 1999; Khaliq et al., 2003) and *in vivo* (Shin et al., 2007), the PC model recorded from soma showed spontaneous *simple spike* generation at rest and increased its discharge frequency upon depolarizing current injection. With high injected currents (>2.2 nA), the model switched to *complex bursting*, characterized by repetitive high-frequency bursts with marked adaptation corresponding to the Ca^2+^-Na^+^ spike bursts observed experimentally (McKay and Turner, 2005; Kim et al., 2013) (Figure 2A; see Movie in Supplementary Material). Upon hyperpolarizing current injection, the model showed sagging inward rectification followed by rebound excitation once the stimulus pulse was terminated. This process involved H channel activation/deactivation and the LVA channel deinactivation/activation generating rebound bursting (Figure 2B). Finally, in response to double-ramp protocols, the model showed a typical V-shaped spike amplitude adaptation (Figure 2C) (Williams and Häusser, 2002). Therefore the model reproduced the fundamental electroresponsive properties of PCs measured using somatic recordings.

When the PC model was injected with step currents (100 pA steps), the steady-state intensity-frequency (F/I) relationship measured from soma was close to linear up to around 300 Hz (Figure 2D), closely matching that measured experimentally *in vitro* in acutely dissociated PCs (Khaliq et al., 2003). When the PC model was injected with a double-ramp current (ranging from 0 to 1.6 nA and back to 0 in 2 s), the instantaneous firing rate started around 64 Hz, reached a maximum of 296 Hz and then decreases to 33 Hz before returning to the steady spontaneous firing frequency of 44 Hz (Figure 2D). Moreover, the spike rate was different depending on whether the ramp current was increasing or decreasing. In all respects, the ramp F/I relationship was similar to that reported experimentally (Williams and Häusser, 2002). Thus, both step and ramp F/I relationships in the model quantitatively reproduced those recorded experimentally. The spike shape observed in the soma of the PC model during simple firing was similar to that measured from acutely dissociated PCs (Raman and Bean, 1999; Swensen and Bean, 2003) and from PCs in brain slices (McKay and Turner, 2005; Kim et al., 2012). The mean spike height was 22 mV, the mean half width was 0.23 ms and the AHP reached a depth of −62 mV. The spike amplitude was 80 mV from AHP to peak with a 6 mV AHP.

When the PC model was injected with step currents higher than 2.2 nA, the response changed from simple firing to complex bursting (Kim et al., 2012). Complex bursting was characterized by calcium spikes triggering sodium bursts and its repetition reflected the cyclic raise and decay of intracellular Ca^2+^ concentration driven by activation of Ca^2+^-dependent K^+^ channels (see below) (Edgerton, 2003; McKay and Turner, 2005; Womack, 2010; Hosy et al., 2011). Complex bursting, which closely resembled that measured experimentally (cf., McKay and Turner, 2005; Kim et al., 2012), consisted of 6 burst/s with spike frequency of 250–300 Hz, in which 4 fast spikes showing increasing size were followed by about 20 spikes with decreasing size followed by a pause and a full-blown Ca-spike repriming the whole process.

### Spontaneous firing and conditions for bistability

**Figure 3:**
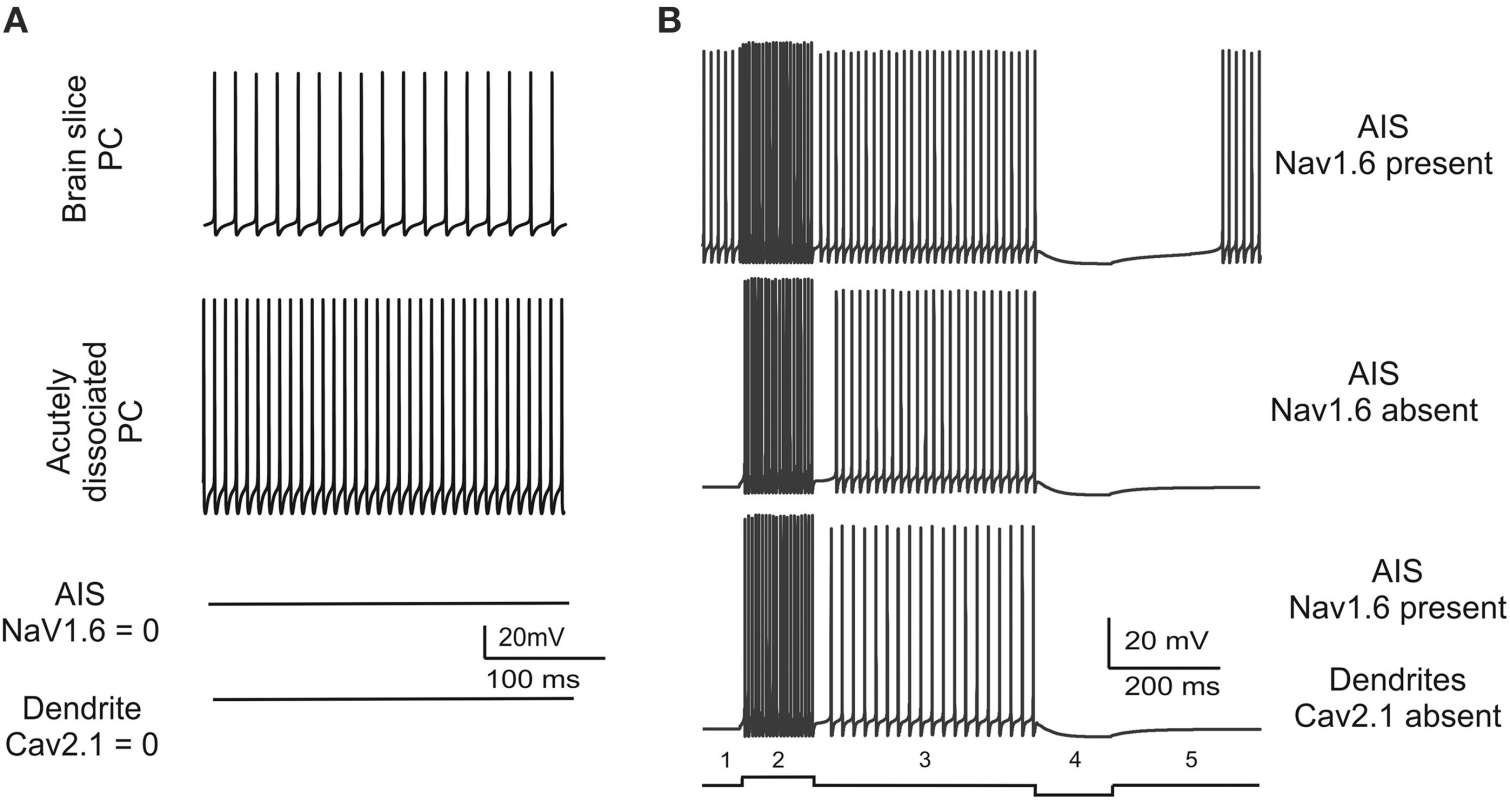
**Spontaneous firing and conditions for bistability. (A)** The traces show model spontaneous firing and its changes in different experimental conditions. The model is set to reproduce a basal 35.5 Hz firing frequency similar to that observed in brain slice recordings. Spontaneous firing frequency increases when dendrites are severed reproducing a pattern observed in acutely dissociated PCs. Spontaneous firing is abolished either with Nav1.6 block in AIS or with Cav2.1 block in the dendrites. **(B)** The traces show that, in the PC model starting from spontaneous firing (1), spike frequency increases upon positive current injections (0.2 nA) (2), recovers to basal level when current injection is terminated (3), goes to zero upon negative current injection (−0.1 nA) (4), recovers to basal level after current injection is terminated (5). The same current steps have different effects when Nav1.6 is switched-off in the AIS or Cav2.1 is switched-off in the dendrites revealing *bistability*. In these latter cases there is no spontaneous firing at rest (1), firing elicited by positive current injection is maintained even when current injection is terminated (“upstate”; 2, 3), firing is persistently switched-off even after negative current injection is terminated (“downstate”; 4, 5).

The consistency of spontaneous firing in the model with that observed in real PCs was attested by three observations (Figure 3). First, the PC model generated regular spontaneous firing which starts at 50 Hz and stabilize around 35.5 Hz in simulation of at least 1 min (Figure 3A). This is similar to spontaneous firing of PCs *in vivo* and *in vitro* (Foust et al., 2010; Palmer et al., 2010), which changes with animal age between 20 and 75 Hz (Khaliq et al., 2003) and lays in the 40–70 Hz range at P90 (McKay and Turner, 2005). Secondly, in the model, switching off the AIS Na channels prevented spontaneous firing (Figure 3A). The AIS is thought to be the site of action potential generation in PCs and also to be responsible of sustaining spontaneous firing. Actually, disrupting the anchoring of Na channels to AIS membrane prevents spontaneous firing and impairs PC excitability (Zonta et al., 2011; Xiao et al., 2013). Thirdly, a protocol was designed to verify whether the dendritic tree of the model contributed to spontaneous firing. It has been hypothesized that this could occur though the background calcium current generated by dendritic P-type channels (Llinas and Sugimori, 1980a,b). However, when the dendritic tree of the model was eliminated, spontaneous firing showed a remarkable frequency increase and the spike amplitude increased as well (Figure 3A). These counterintuitive changes reflected the raise in input resistance, as also observed in experiments on acutely dissociated cells, in which soma and axon were preserved but dendrites were severed (Khaliq et al., 2003). However, the removal of Cav2.1 from the dendrites (but not from other model sections) blocked spontaneous firing much like the removal of Na channels from AIS (Figure 3A).

In summary, results reported in Figure 3A show that spontaneous firing is an intrinsic property of the PC model sustained by sodium channels in the AIS and by P-type calcium channels (Cav2.1) in the dendrites. When either one of these two channels was missing, pacemaking disappeared and the model became *bistable*, i.e., it could be switched between two stable states by an external stimulus. In the PC model deprived of sodium channels in the AIS or of P-type calcium channels in the dendrites, a brief positive (0.2 nA–200 ms) or negative (−0.1 nA–200 ms) current injection was able to toggle the PC model between the *up-state* and the *down-state* (Figure 3B), as observed in certain functional conditions (Loewenstein et al., 2005; Schonewille et al., 2006; Rokni et al., 2009).

### The mechanisms of simple spike generation and propagation

**Figure 4:**
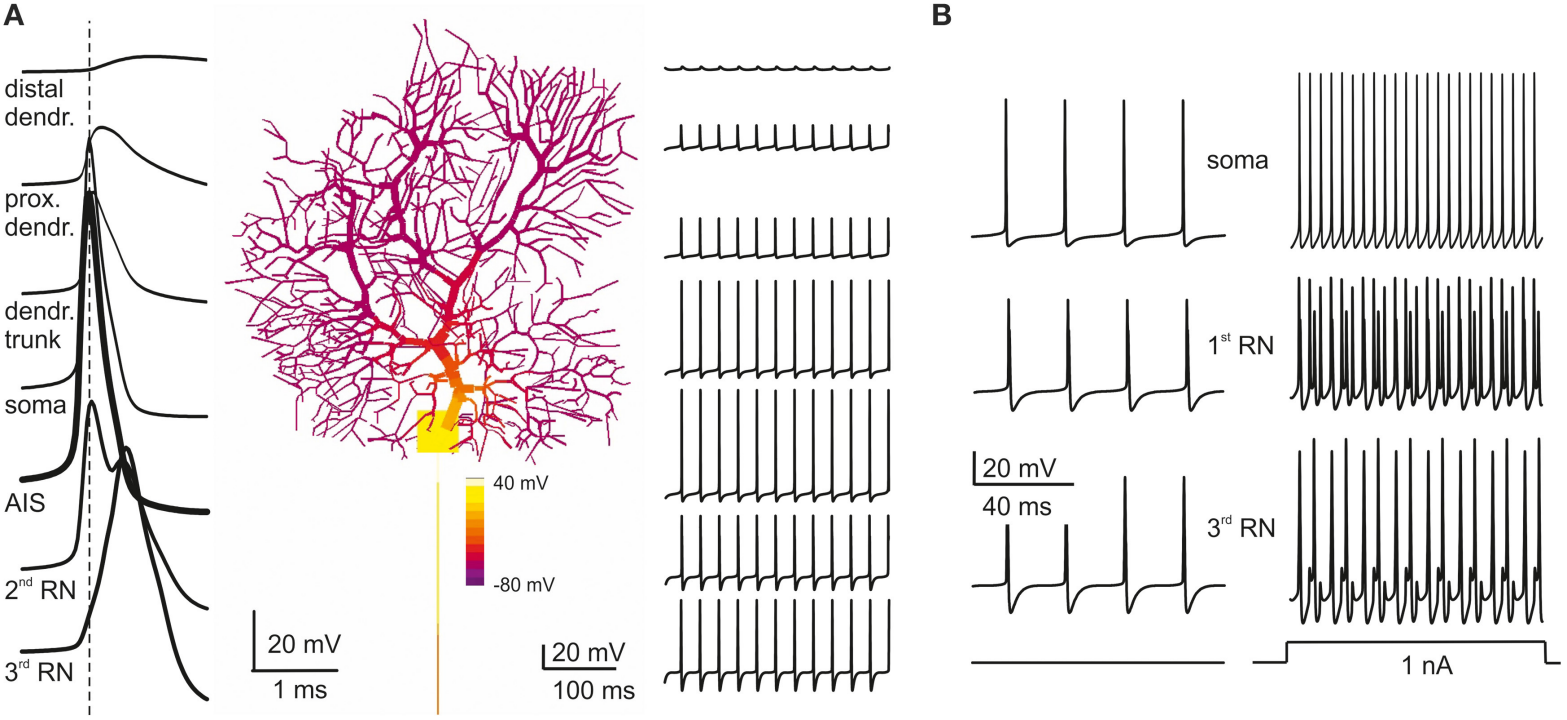
**Action potential generation and propagation. (A)** PC model activity is shown at the time of AIS excitation during spontaneous firing (membrane potential is color-coded onto model morphology). Action potentials traces are shown at different locations (single spikes on the left, a brief spike train on the right). Action potentials are generated in the AIS, back propagate into the soma and forward propagate into the axon, such that the AIS spike anticipates those in the other model sections (take the vertical dashed line for reference). When action potentials reach the dendritic tree, their amplitude decays sharply. **(B)** During *spontaneous firing*, spike frequency remains the same from soma to 3rd RN showing that the axon transmits low frequencies reliably. In response to *current injection* (>1 nA), the PC model generates regular full-blown spikes at a frequency over 200 Hz in the soma and AIS but, in 3rd RN, spikes generation becomes relatively independent from AIS thereby limiting transmission at high frequencies.

A fundamental observation characterizing PC electroresponsiveness is that action potentials recorded either extracellularly (Llinas and Sugimori, 1980a,b) or intracellularly (Stuart et al., 1997; McKay and Turner, 2005) decrease progressively from full-blown spikes to heavily filtered spikelets while moving from somatic toward the farthermost dendrites (see Movie in Supplementary Material). This property was reproduced by the model and reflected the disappearance of Na channels (Fry et al., 2007) while moving from soma into the dendrites (Figure 4A) combined with dendritic passive properties limiting current back-propagation (Roth and Hausser, 2001).

Relevant information on the mechanism of spike generation and propagation has recently been provided by using patch-clamp recordings, showing that spikes rise first in the axon initial segment (AIS) and then propagate antidromically to the soma and orthodromically through RNs and the axonal branch. In the model, the spikes generated in the AIS preceded by about 0.1 ms those in the soma and first RN. The axon (from AIS to 3rd RN) conducted action potentials at 0.73 m/s, close to the natural conduction velocity (0.77 m/s; Clark et al., 2005) (Figure 4A). The reliability of transmission was monitored by determining the presence of full-blown spikes at the 3rd RN (Figure 4B). Spikes generated in the AIS could be elicited at over 300 Hz but then they were reliably transmitted along the axon only below 200 Hz, as observed experimentally (Figure 4B; Monsivais et al., 2005) provided that specific K channels were placed in RNs (see below).

### Axo-somatic currents during simple spike generation and propagation

**Figure 5:**
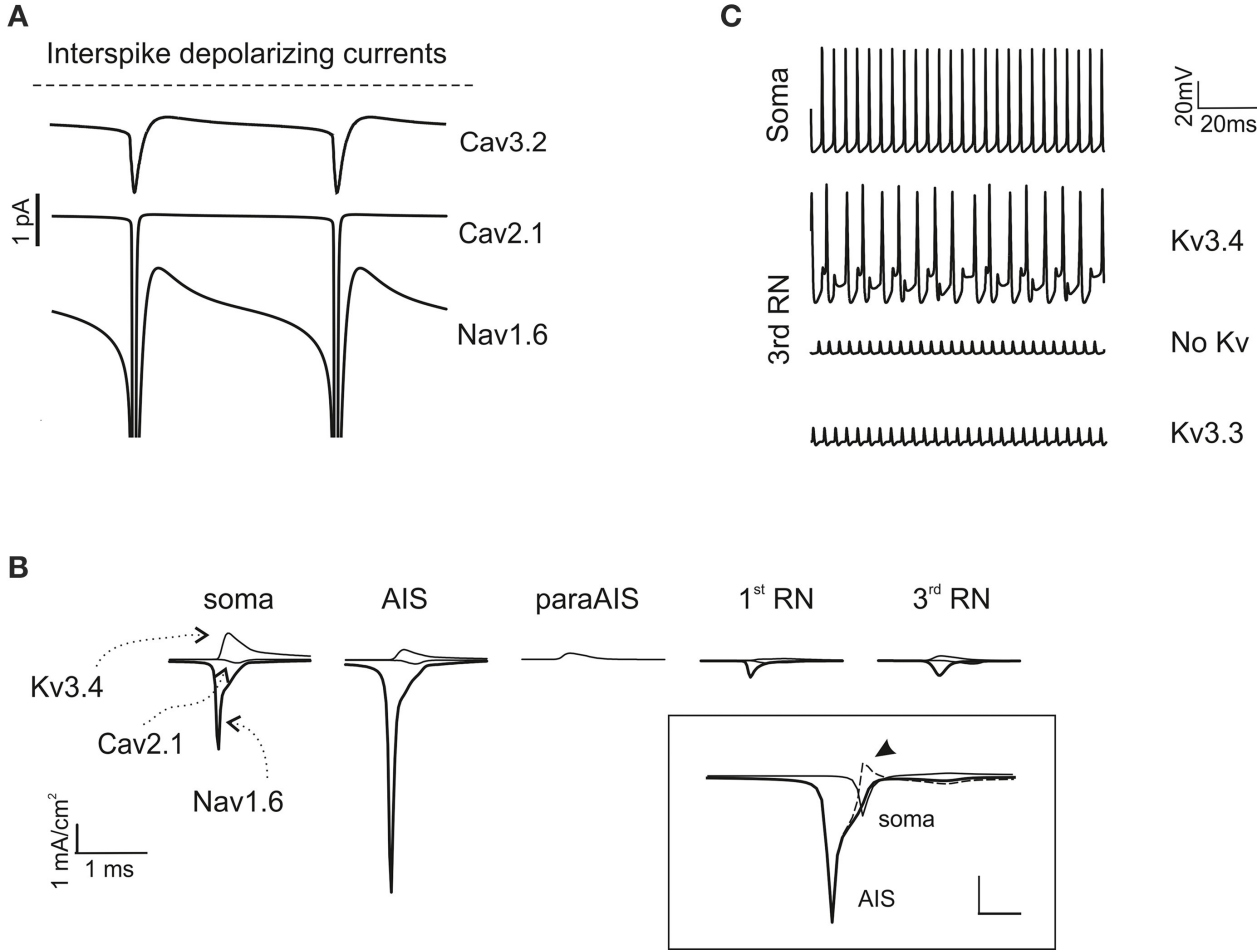
**Ionic mechanisms of action potential generation. (A)** The traces show the major inward depolarizing currents generated during the interspike interval, which are capable of sustaining spontaneous firing. These include Cav2.1, Nav1.6 and Cav3.2. **(B)** Axonal currents involved in action potential generation during spontaneous firing. Note that Nav1.6 current is larger in the AIS than in the soma and RNs. Note also absence of Na and Ca currents in the paraAIS. The *inset* shows AIS and somatic Nav1.6 currents along with their algebraic difference (dashed line): the peak somatic current occurs nearly 1 ms after the AIS current (the arrow indicates the excess of somatic Na current). **(C)** The traces show the effect of Kv3.3 and Kv3.4 on action potential generation at the 3rd RN. Note that Kv3.3 (at double concentration that Kv3.4) is poorly effective, while Kv3.4 effectively controls spike afterhyperpolarization and reduces firing frequency.

As noted above, in the model, the currents needed to generate autorhythmic firing included currents generated by AIS Na channels and dendritic Ca channels. Actually, a persistent Nav1.6 current as well as Cav2.1 and Cav3.2 currents appeared in the interspike interval during spontaneous activity (Figure 5A). The currents generated by somato-dendritic Ca channels diffused to the AIS, contributing to generate the depolarizing drive required to activate spontaneous firing.

In the AIS, a strong regenerative current appeared during spike generation (Figure 5B), akin with the high concentration of Na channels in AIS and the observation that PC spikes cannot be generated when the axon is severed (Nam and Hockberger, 1997). A low concentration of Na^+^ channels in the soma and dendritic trunk was nonetheless required to counterbalance the low impedance of these PC compartments compared to the axon, thus preventing current loss and stabilizing the spike generation mechanism (Figure 5A). Moreover, a back-propagation of Na current from soma to AIS, which occurs given the delay in somatic spike generation, was also probably involved in reinforcing the AIS spike generation process (Figure 5B inset).

As noted above, the highest Na current transient occurred in the AIS, followed by soma and RNs. Despite their small size, Na currents in RNs were sufficient to ensure appropriate spike regeneration and propagation due to the high axonal input resistance. The Ca and K currents were comparatively smaller but could also play a relevant role. In particular, K channel activation in soma, AIS and RNs controlled spike AHP and therefore the duration of the interspike interval. Experiments have shown a major expression of Kv3.4 in the soma (Martina et al., 2003) and of Kv3.3 in the axon (Chang et al., 2007). While both channels have inactivation and show A-type properties, unfortunately available Kv3.3 models do not incorporate the inactivation mechanisms normally provided by PKC-dependent phosphorilation. Therefore, we placed the homologous Kv3.4 in the PC model axon, also considering that this channel could regulate spikes generation in a previous single compartment model (Khaliq et al., 2003). KV3.4 ensured that RNs followed AIS at frequency up to 200 Hz but then limited the maximum axonal transmission frequency (see Figures 3B, 5C). Placing the available model of Kv3.3 in RNs (even at a density 100-time higher than Kv3.4) did not provide efficient spike frequency control (Figure 5C).

### Transition from simple firing to complex bursting

**Figure 6:**
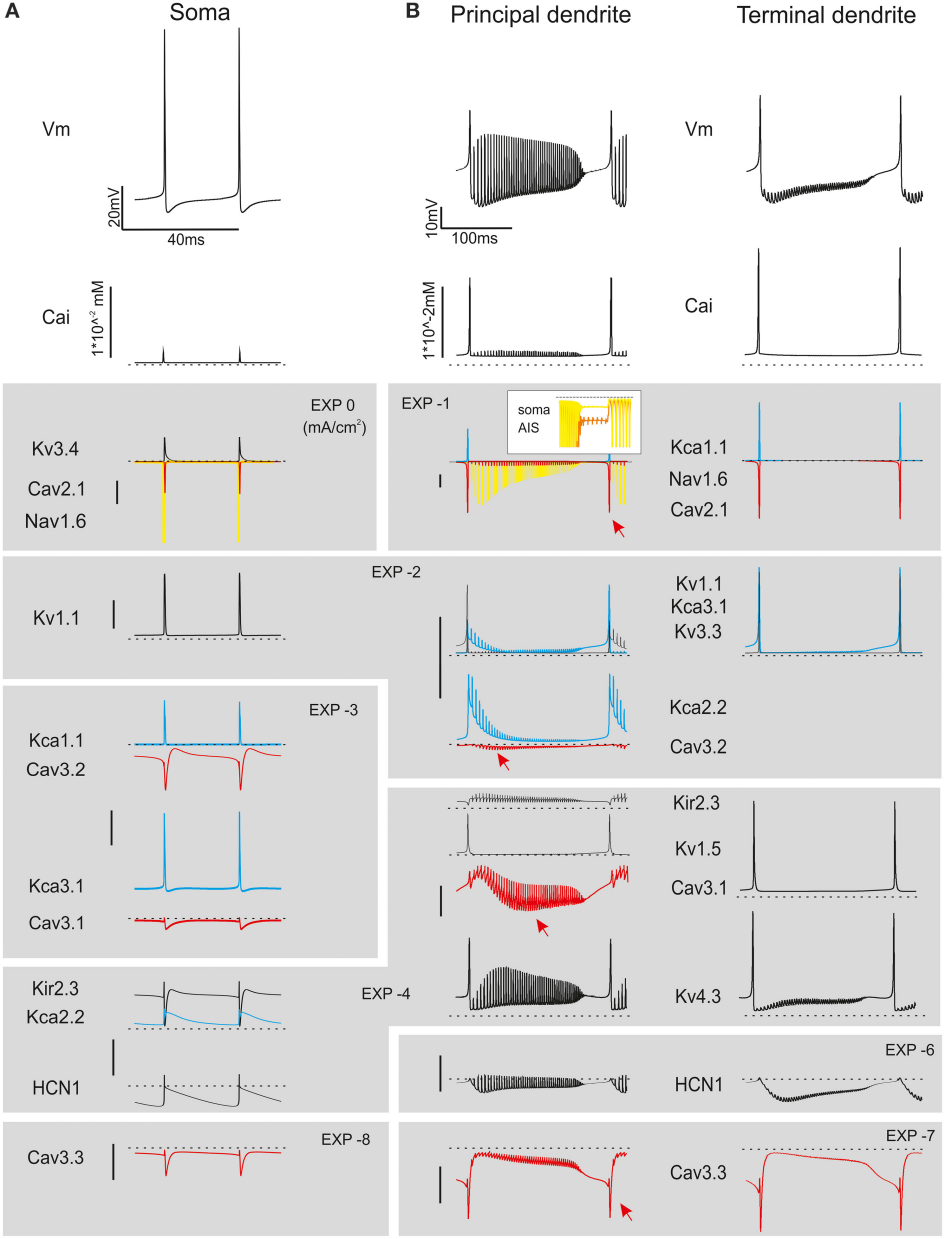
**Ionic currents during simple spike firing and complex bursting**. Membrane potential, calcium concentration and ionic currents during simple spike firing and complex bursting. **(A)** Traces show ionic currents in the soma during spontaneous firing. **(B)** Traces show ionic currents in the principal dendrites (>8 μm) and terminal dendrites (<3.5 μm) during complex bursting (2.2 nA current injection). It should be noted that several spike-related currents are larger in terminal than proximal dendrites reflecting the dendritic generation mechanisms involved in complex bursting. The inset shows soma and AIS Nav1.6 current that can back-propagate into the dendrites sustaining complex bursting. Both in A and B, colors are used to better identify certain currents (yellow for Nav1.6, red for Ca currents, blue for Kca currents). Arrowheads point to the peak of LVA currents. Currents are grouped and ordered from largest (top) to smallest (bottom) according to a logarithmic scaling of calibration bars.

During simple firing, the cycle of spike depolarization and repolarization proceeded regularly, regulated by the numerous K currents of the PC model generating a robust AHP and removing Na channel inactivation (Figure 6A). However, beyond a certain level of current injection, firing frequency was so high that Na channel repriming by AHP in the AIS became incomplete generating a vicious cycle: lower Na current caused smaller spikes, smaller K channel activation, smaller AHP, smaller Na channel de-inactivation and so forth, until spikes faded and the burst terminated. Then, the reduction of K-driven repolarisation allowed full activation of Ca currents in the dendrites generating a Ca spike concluding the burst (Figure 6B; see Movie in Supplementary Material). The Ca spike was initiated by LVA currents and culminated with a large HVA-driven spike reflecting the different activation range of these channels. The engagement of LVA currents showed a typical pattern reflecting the biophysical properties and distribution of ionic channels, with Cav3.1, Cav3.2, and Cav3.3 currents showing maximal activation at the beginning, the middle and the end of the burst, respectively.

**Figure 7:**
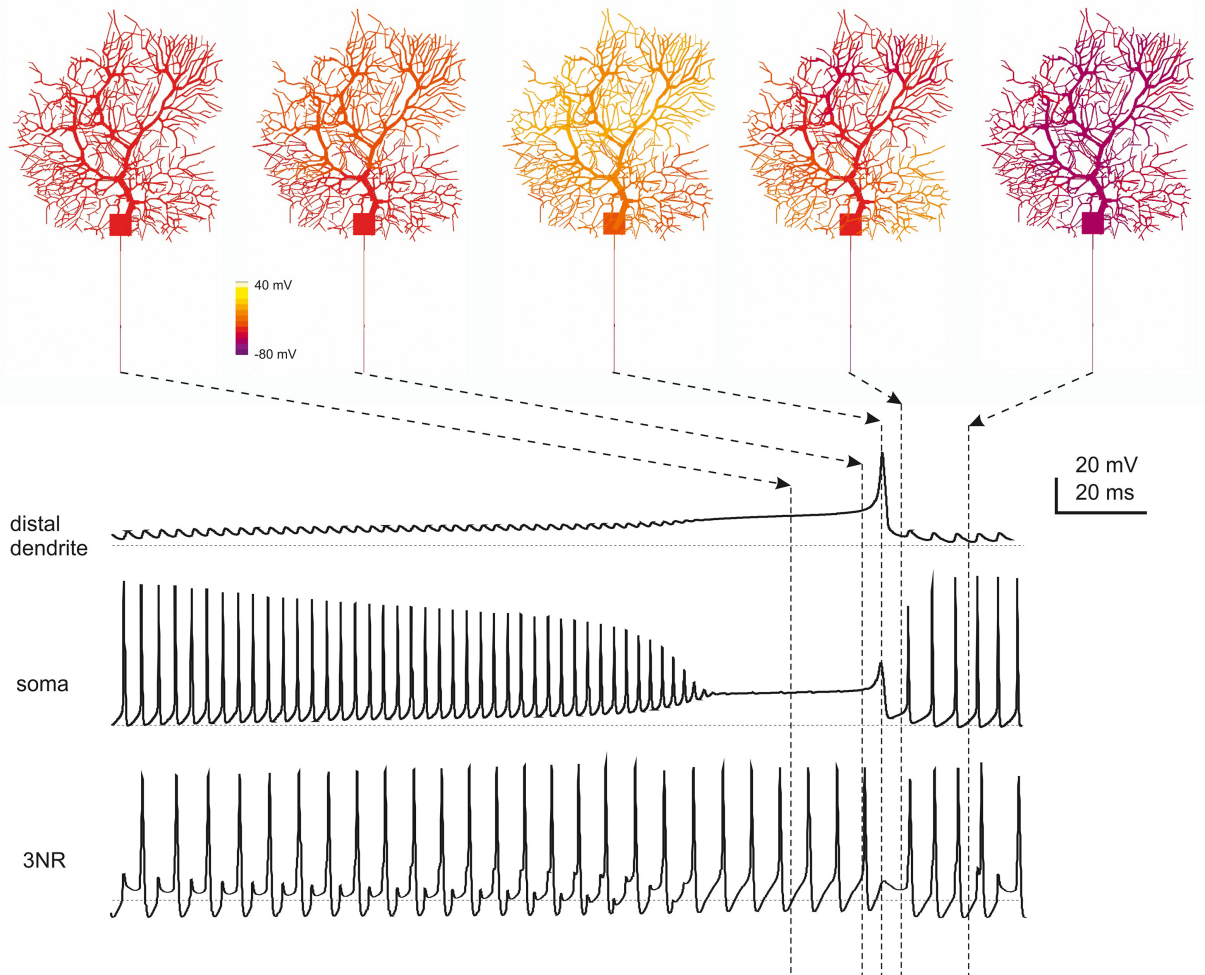
**Spatiotemporal dynamics of PC firing during complex bursting**. The panels on top show screenshots of PC membrane potential observed at different times during complex bursting (membrane potential is color-coded onto model morphology). Arrows point to the corresponding time on the traces representing membrane potential recorded in distal dendrites, soma and 3rd RN. By looking at the sequence of screenshots from left to right it appears that, at the end of the spike burst, the PC model depolarizes starting from distal dendrites before the depolarization invades the whole dendritic tree. A large Ca spike is the most relevant depolarizing event in terminal dendrites, while fast Na spikes are most evident in AIS. In the 3rd RN, there is no firing pause during the dendritic Ca spike.

During complex bursting, Ca currents became larger in the dendrites than in axo-somatic compartments, so that the control of action potential generation temporarily passed to the dendrites (Figure 7) reproducing a property typical of PCs (McKay and Turner, 2005; Kim et al., 2013). Interestingly, during complex bursting, intracellular diffusion of dendritic depolarizing currents into the axon was strong enough to activate action potentials in RNs, even when the main AIS generator was silenced by Na channel inactivation. This effect, which remains to be tested experimentally, could imply the intervention of secondary spike generation mechanism in RNs at high discharge regimes.

### Model robustness

Model robustness was assessed in three different conditions. First by determining the parameter range allowing to maintain *canonical* PC properties, secondly by generating PC variants, and thirdly by evaluating the impact of current injection in the dendrites to emulate synaptic inputs.

#### Parameter changes in the canonical PC

**Figure 8:**
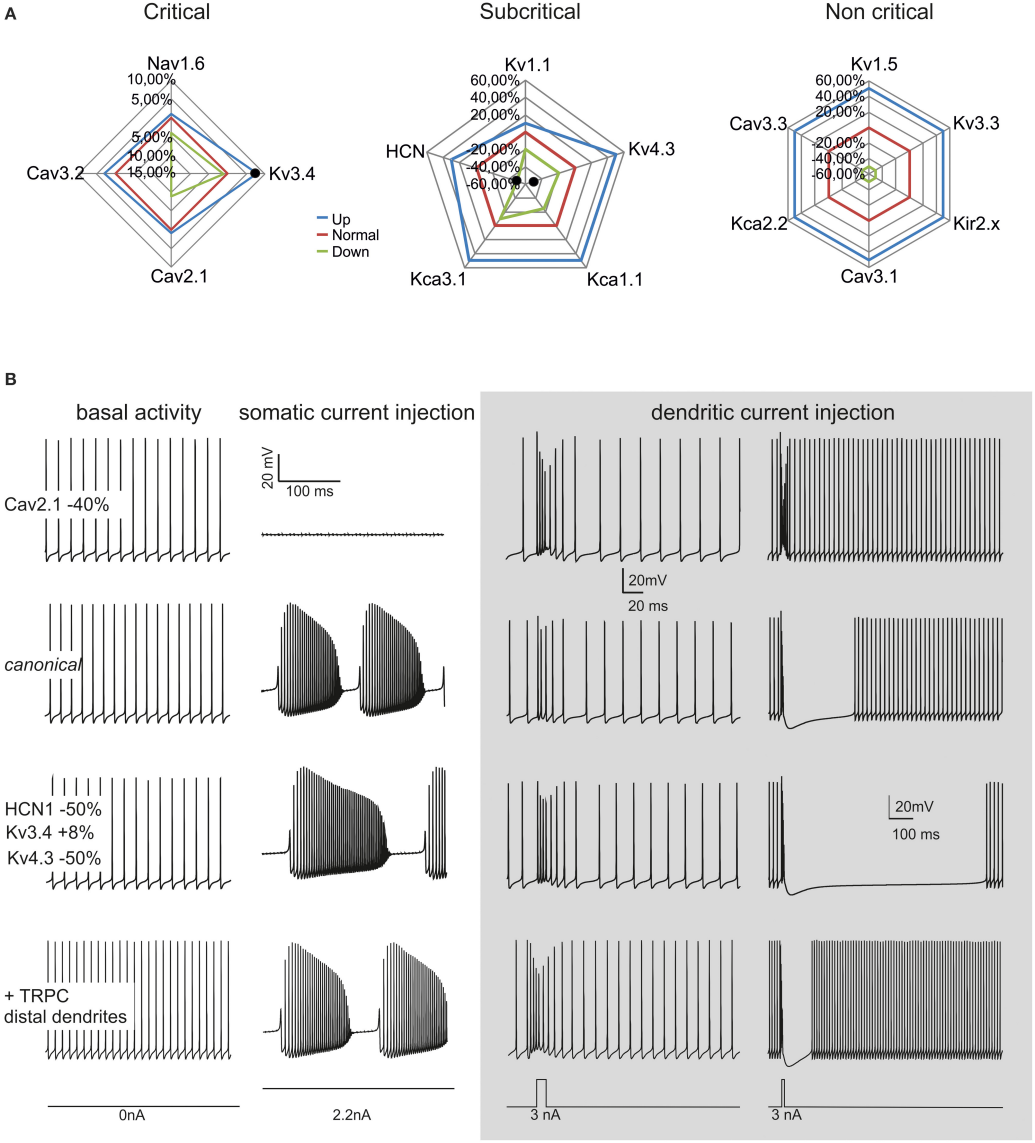
**Model robustness**. Robustness of the *canonical* PC neuron and variant PC firing patterns. **(A)** The graphs show the fluctuation ranges of maximum ionic channel conductances needed to maintain the model within PC physiological response limits. The channels have been grouped into *critical*, *subcritical*, *non-critical*. The lines indicate the standard (*red*), minimum (*green*) and maximum (*blue*) values allowed. **(B)** The model was changed to simulate a CF-PC (−40% Cav2.1), a P-PC (HCN1 −50%, Kv3.4 +8%, Kv4.3 −50%) and a Z- PC (TRP channels in the dendrites). The traces show spontaneous firing and complex bursting in the different cases. *Gray panel:* model responses to dendritic current injection (3 nA/10 ms in terminal dendrites, 3 nA/30 ms in dendritic trunck) in the different cases. Note the similarity of these responses with those typical generated by parallel fibers and climbing fibers activity.

Model robustness was assessed by evaluating the range of variation allowed for each ionic conductance in order to maintain model electroresponsiveness within the experimental values measured for spontaneous frequency, F/I relationship and complex bursting. The ionic channels were divided into three “classes” depending on the maximum conductance variations (Δg_max_) allowing all the features to remain within physiological limits (see Methods and Figures 4, 5): *critical* (Δg_max_ < ±15%), *subcritical* (±15% < Δg_max_ < ±30%), *non-critical* (Δg_max_ > ±50%). Critical channel included Nav1.6, Cav3.2, Cav2.1, Kv3.4, subcritical channels included Kv1.1, Kv4.3, KCa1.1, KCa3.1, HCN, non-critical channels included Kv1.5, Kv3.3, Cav3.1, Cav3.3, KCa2.2, Kir2.x. The increase or decrease in maximum ionic conductances generated upper and lower boundaries reported on the graphs of Figure 8. These graphs showed that the model was well balanced around the middle of the variation boundaries for all ionic conductances used.

The robustness analysis was extended to assess how the four critical channels affected spike generation depending on PC model sections (not shown). Nav1.6 proved more important for firing in the soma than AIS. A 25% Δg_max_ reduction in the soma prevented spontaneous firing. This suggests that somatic sodium channels boost the spike whether the depolarizing current comes from the AIS or from the dendrites, whereas the AIS sodium channels acts as effective spike generators. A similar yet opposite role was played by the main potassium channel in the PC model, Kv3.4, in a way that the increase in somatic Kv3.4 blocked PC firing. The calcium channels showed a marginal impact on spikes generation, with variation in the order of 1–2% for channel reductions in the soma and AIS. No impact on spikes generation was observed for any of these channels when their conductance was changed in the rest of the axon.

#### PC firing variants

Recently, variant PCs with high basal discharge frequencies were observed and related to Zebrin strips (about 60 Hz in Z+ PCs and about 100 Hz PCs in Z− PCs; Zhou et al., 2014). Although synaptic bombardment from parallel fibers *in vivo* could raise the PC basal frequency, TRPC3 channels in the dendrites may also play a distinctive role. Indeed, the PC model could easily generate high basal frequencies (e.g., 100 Hz) by placing TRP channels in the dendrites (Figure 8B). Two other variant PC firing patterns were identified based on responses to synaptic stimulation (see below), named “continuously firing” and “pausing” PCs (CF-PCs and P-PCs; Yartsev et al., 2009). Compared to ordinary PCs, CF-PCs do not make relevant pause after PF stimulation, while P-PCs make a pause longer than normal. CF-PCs were successfully modeled with a diffused HVA Ca^2+^ channel reduction (−40% Cav2.1), while P-PCs were modeled with combined channel changes affecting H-channel (−50% HCN1) and K^+^ channel changes (+8% Kv3.4, −50% Kv4.3).

#### Dendritic current injection

Although a full investigation of PC synaptic inputs goes beyond the aims of this paper, PF and CF activity was emulated by dendritic current injection. The model generated typical burst-pause responses depending on the duration and location of current injection. Burst-pause responses imitating a parallel fiber input were obtained by short (10 ms) step-current injection into terminal dendrites, while responses imitating complex spike generated by climbing fiber input were obtained with longer (30 ms) step-current injection into the dendritic trunk (Figure 8B). These responses, which closely resemble those generated in PCs by synaptic activation, could be modulated in the CF-PC and P-PC variants, as explained above.

### Model predictions on ionic channel mutations

**Figure 9:**
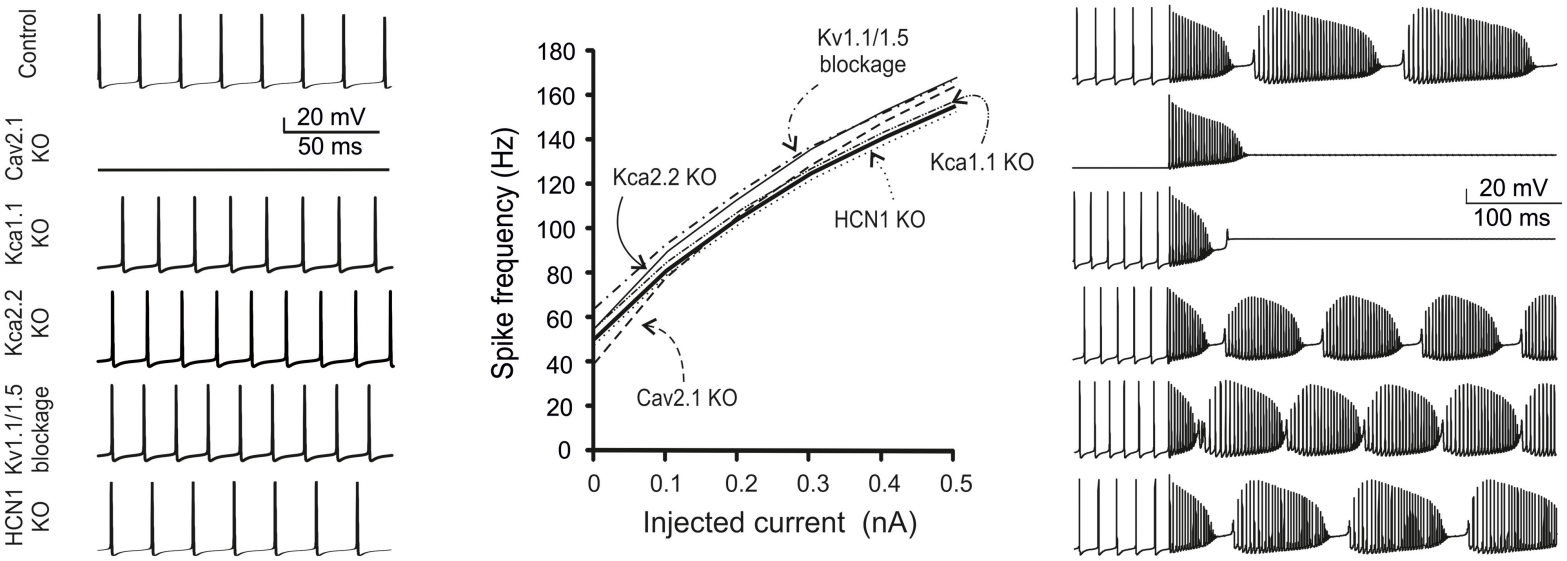
**Ionic channel mutations and modulation in the model**. The figure shows simulations of changes in firing pattern caused by gene mutations or pharmacological inhibition of PC ionic channels. Traces show model spontaneous firing (left) and complex bursting (right; 2 nA current injection). The plot shows the frequency-intensity relationships in the different cases. From top to bottom: Nav1.6 switch-off fully abolished firing; Cav2.1 switch-off prevented spontaneous firing and complex bursting; Kca1.1 switch-off prevented complex bursting; Kca2.2, Kv1.1, and Kv1.5 switch-off caused a slight increase in spontaneous and evoked firing; HCN1 switch-off did not alter spontaneous and evoked firing remarkably.

Simulations were also performed to evaluate the impact of ionic channel mutations. We considered both critical and subcritical/non-critical channels (Figure 9).

#### Nav 1.6 KO

Nav1.6 switch-off totally abolished firing in the model. Notably, in Nav1.6 KO mice, spontaneous firing was maintained by compensatory expression of Nav1.1/1.2 isoforms (Lorincz and Nusser, 2008) and this conditions was not simulated. The effect of disrupting the anchoring of Na channels to AIS membrane (Zonta et al., 2011; Xiao et al., 2013) has been successfully simulated leading to suppression of spontaneous firing (see Figure 3).

#### Cav2.1 KO

This KO is particularly important, since Cav2.1 is the main calcium channel of the PC, is located along the entire neuron and provides the main source of calcium entry into the dendrites (90%). The KO of this channel prevented the PC model from generating spontaneous firing (Mark et al., 2011). Moreover, Cav2.1 KO prevented complex bursting in the PC model by reducing the dendritic depolarising drive.

#### Kca1.1 KO

Kca1.1 has been reported to counterbalance calcium entry through Cav2.1 and to co-localize with it (Indriati and Kamasawa, 2013). With Kca1.1 KO, in the PC model spontaneous firing was not compromised but rather it became slightly faster, as expected from a stronger depolarizing action of Cav2.1. Moreover, Cav2.1 KO prevented complex bursting in the PC model by impairing spike calcium-dependent AHP.

#### Kca2.2 KO

The Kca2.2 KO made the PC more excitable (Hosy et al., 2011) and spontaneous and evoked firing was even faster than with Kca1.1 KO. However, unlike with KCa1.1 KO, with Kca2.2 KO complex firing in the model was not abolished.

#### Kv1.1 and Kv1.5 KO

The double KO of Kv1.1 and Kv1.5, which are known for their ability to prevent dendritic hyper excitability (McKay et al., 2005), resulted in a slight increase in spontaneous and evoked firing.

#### HCN KO

The HCN current is very small (10 pA), compared to those generated by calcium, sodium or potassium channels, but it is important for post synaptic integration. Actually, HCN KO in the PC model maintained spontaneous firing almost unchanged, as it occurs in real PCs (Biel and Wahl-Schott, 2009).

These results conform to the effect of genetic KO in mice providing an additional source of validation to the model.

## Discussion

The main result of this PC model is to provide a unified hypothesis on action potential processing based on localization and gating of ionic channels. In the model, simple spikes were generated in the AIS and protracted spike discharge was stabilized in the soma through Na-dependent mechanisms. Dendritic Ca currents contributed to sustain spontaneous discharge and complex bursting. At high discharge regimes, spike frequency was filtered in the axon by K channels. The model did not show bistability, but transitions between *up* and *down* states could be obtained by down-regulating Ca and Na currents. The model thus demonstrates that the axon—including the AIS, paraAIS and RNs—is essential to explain action potential processing in PCs reconciling the large set of results reported experimentally. Properties, implications and limits of the model are discussed.

### The PC canonical model

The model summarizes the prototypical properties of a PC and can therefore be considered a *canonical model* (Segev et al., 1989). The present setting of maximum ionic conductances provided an effective solution but their changes within limited ranges did not prevent model functioning (cf., Achard and De Schutter, 2006). Variants of this canonical model accounted for differences in the basic responses to synaptic stimulation identified as CF-PCs and P-PCs (Yartsev et al., 2009). Moreover, the increased basal firing frequency typical of Z- PCs was reproduced by inserting TRP channels in dendrites (Zhou et al., 2014). Combinations of these properties may also account for PCs with repeated switches between high-frequency UP-states and the DOWN state (Cheron et al., 2014). Therefore, the PC *canonical* model is just one of the possible solutions giving raise to documented PC firing patterns. The impact of multiple conductance combinations may be further investigated by generating populations of PC models through optimization strategies based on genetic algorithms (Druckmann et al., 2011, 2013).

Model morphology was based on a well-established reconstruction taken from a guinea pig PC (Rapp et al., 1994) and was implemented by adding the axonal compartments. This extension to previous models (De Schutter and Bower, 1994a,b) was needed to generate the complex action potential processing typical of PCs. The potential impact of PC dendritic morphology—which slightly varies from cell to cell, between cerebellar subdivisions (Anwar et al., 2010; Nedelescu and Abdelhack, 2013) and during ontogenesis and phylogenesis—on spike generation remains to be tested.

The model incorporated 15 voltage-dependent ionic channel subtypes updating the more limited set of channels previously used to model PCs (De Schutter and Bower, 1994a,b). Apparently, not all channels proved equally critical for action potential processing and could partially compensate one for each other. It should be noted that subcritical and non-critical channels were mostly dendritic. Thus, probably, their actual role could not be fully demonstrated by using somatic current injection, but it could be determined by using synaptic inputs (e.g., Solinas et al., 2007a,b).

### Model construction strategy and biophysical constraints

Important data for model construction came from immunohistochemical indications about ionic channel location. Na channels were concentrated in AIS and Ranvier nodes but were also present in the soma, HVA and LVA Ca channels were more abundant in dendrites than elsewhere, K and K-Ca channels were specifically distributed among axon, soma and dendrites with the remarkable absence of any K channels from the AIS and enrichment in paraAIS. This asset proved critical as the reduction or absence of any of these channels compromised basic properties of PC electroresponsiveness. Most notably, reduction of Na channels in AIS prevented spike generation, reduction of Na channels in RN prevented spike propagation, reduction of Na channels in the soma prevented sustained firing. Likewise, reduction of HVA and LVA channels in the dendrites prevented spontaneous firing and complex bursting, and reduction of K channels in the RNs prevented firing frequency filtering.

A main constraint for setting ionic channel densities was provided by the total amount of Na channels, which matched the maximum Na current measured from the soma of dissociated PCs under VC (Swensen and Bean, 2003). Then the relative Na channel density in the AIS, axon and initial dendrite was set according to results obtained in axonal and dendritic whole-cell recordings (Bender and Trussell, 2009, 2012; Bender et al., 2012). After Na channel densities had been fixed, corresponding settings in other currents allowed to obtain appropriate action potential shape and frequency. A second constraint was provided by Ca channels densities, which, along with those of dendritic K channels, were set to generate complex bursting. A third constraint was provided by coupling K to Na currents in the axon in order to respect axonal transmission speed and frequency filtering properties.

Consistently, the Na, Ca and K currents generated during simple spike firing were larger in soma than dendrites, while the Ca and K current generated during complex bursting were larger in dendrites than soma. Beyond this, the model provides theoretical predictions on local current flows.

### Model complexity and effectiveness

A striking aspect of the PC model is to incorporate 15 voltage-dependent ionic channels expressing a complex blend of nonlinear membrane dynamics (see Figure 1). This channel set expands that adopted previously (e.g., see De Schutter and Bower, 1994a,b), which included just a Na channel, an HVA and LVA Ca channel, and K channels of KV-type A-type and KCa-type. Moreover, the PC model, by implementing the axon, included even more compartment than previous ones. The question is to what extent this new detailed description is needed for understanding PC electroresponsiveness or rather, weather a simplified morphology or a limited number of channels would be sufficient (for example, a compressed PC model was recently shown to properly respond to synaptic inputs, Marasco et al., 2013).

The apparent advantage of compartmentalization was to allow separated control over pacemaking, simple firing and complex bursting. In particular, axonal compartmentalization improved independent control over spike generation and transmission. The model also hinted at the diversity of PC ionic channels (see Figures 7, 8). Critical (Nav1.6, Cav3.2, Cav2.1, Kv3.3) and subcritical channels (Kv1.1, Kv4.3, KCa1.1, KCa3.1, and HCN1) played distinctive roles in pacemaking, simple firing and complex bursting. The minor impact of non-critical channels (Kv1.5, Kv3.3, Cav3.1, Cav3.3, KCa2.2, and Kir2.x) was related to their dendritic location, making them poorly effective in axo-somatic control of firing. Nonetheless, these channels are likely to play a distinctive role in synaptic integration and plasticity. Therefore, the PC has a large potential for firing pattern regulation, which has only been partially explored here but may be fundamental to explain PC functions during network activity (see for example SK2 channel changes: Belmeguenai and Hosy, 2010; Hosy et al., 2011; Ohtsuki et al., 2012).

Although an extensive biophysical investigation would be required to unveil the implications of specific ionic channel gating properties (see Figure 1), some distinctive effects were immediately evident. Nav1.6 channels, with their transient persistent and resurgent components, were related to the need of generating both simple and complex spikes (Lewis and Raman, 2014). Cav3.2, Cav3.1, Cav3.3 LVA channels, through their gradient of activation and inactivation voltages (from more to less negative) and time-constants (from faster to slower), could fine-tune the rate of growth, intensity and duration of complex bursts. Cav2.1 HVA channels, by activating almost instantaneously at voltages higher than LVA channels, regulated KCa currents and AHP and intensified the complex burst. The effectiveness of Kv3.4 in controlling axonal spike conduction and frequency filtering supports the hypothesis that appropriate gating of RN K channels (A-type), rather than inactivation of Na channels, were involved (Monsivais et al., 2005; Duflocq et al., 2011; Yang and Wang, 2013). The implications of the variety of K channels expressed in the soma and dendrites (Figure 1B) emerged from their differential involvement in simple firing (Kca1.1, Kv1.1, Kv1.5, Kv3.3) and complex bursting (Kca2.2, Kca3.1, Kv4.3) allowing to regulate spike patterns over different time scales (see Figures 5, 6).

Therefore, although the model is more complex and computationally expensive than previous ones, it provides a closer explanation of biophysical mechanisms and improves control over the multiple electroresponsive properties of PCs.

### Hypothesis on the mechanisms of spike generation and propagation in PCs

The *unified hypothesis* on PC action potential generation formulated by the model can be summarized as follows. (i) AIS Na channels are fundamental for simple firing, while dendritic Ca channels are fundamental for complex bursting. (ii) Protracted spike discharge is maintained by Na channels located in the soma and main dendrites, which contrast charge dissipation into the dendrites and provide reverse charge transfer toward AIS during the late phase of the action potential. (iii) Ca channels in the dendrites generate a window-current that, once transferred to the AIS, sustains PC pacemaking. (iv) Complex bursting is sustained by dendritic Ca currents but also by a persistent/resurgent Na current diffusing from AIS and soma to the dendrites. (v) The maximum output spike frequency can be limited by A-type K channels in the paraAIS and RNs. (vi) The RNs may become secondary action potential generators once the primary generator in the AIS is inactivated, e.g., when receiving Ca currents diffusing from the dendrites during complex bursting.

Thus the models supports the hypothesis that action potential processing in PCs is a distributed mechanism, in which a primary AIS generator is active during sample firing but can be overdriven by secondary generators in the dendrites and RNs during complex bursting. Moreover, in addition to support the Na-dependent hypothesis (Khaliq et al., 2003; Clark et al., 2005; Palmer et al., 2010), the model also provides a clarification for the Ca-dependent hypothesis of spike generation. The original Ca-dependent hypothesis maintained that spike generation in PCs was Ca-dependent (like in the heart) because of the large Ca conductance expressed in the dendritic tree (Llinas and Sugimori, 1980a,b). Indeed, in the model somato-dendritic Ca channels sustain spontaneous firing in addition to generate complex bursting. Finally, the model predicts that axonal frequency filtering cannot be obtained through Na channel inactivation (as proposed by Yang and Wang, 2013) but requires specific K channels in RNs, in agreement with the role predicted for these channels in the paraAIS of motoneurons (Duflocq et al., 2011). Na channel inactivation, can indeed block firing in the AIS during complex bursting but does not prevent spikes from traveling through the axon due to secondary spike generation in RNs. Finally, since bistability turns out to be incompatible with a fully functional PC model, it is suggested to reflect a modulatory state in which Na and Ca conductances are reduced. A reduced Na and Ca channel conductance can be caused by general anesthetics, which favor PC bistability *in vivo* (Schonewille et al., 2006).

### Implications of model properties for PC function

The simulations using dendritic current injection suggest that PCs electroresponsive properties provide the basis to explain synaptic integration: parallel fiber synapses located on terminal dendrites could generate currents diffusing to AIS generating simple-spike burst-pause responses (Steuber et al., 2007), while climbing fiber synapses located on dendritic trunk could engage LVA and HVA channels activating dendritic Ca spikes and resurgent Na currents in AIS (much like as in complex bursting, Khaliq et al., 2003). Inhibitory synapses impinging on the dendrites may locally modify voltage-dependent gating of Ca and K channels regulating the pauses following spike bursts and local synaptic integration (Solinas et al., 2006). Basket cell inhibition may exploit the low presence of K channels in the AIS implementing an ephaptic inhibitory process (Blot and Barbour, 2014) thereby fine tuning AIS activity (Duflocq et al., 2011). The impact of specific synaptic mechanisms, along with the effect of complex spatio-termporal patterns of excitatory and inhibitory synaptic activity, remain to be modeled.

It should also be noted that decremental simple-spike diffusion from AIS to dendrites is consistent with a mechanisms of coincidence detection through local synaptic interactions rather than spike back-propagation, as predicted by main theories on parallel fiber—Purkinje cell LTD (Marr, 1969; Mittmann and Häusser, 2007; D'Angelo, 2014). Finally, low-pass filtering of spike frequency in the axon may serve to reduce energy expenditure and to prevent over-inhibition of DCN cells (Monsivais et al., 2005; Yang and Wang, 2013).

## Summary and conclusions

Since the model quantitatively reproduced simple firing as well as complex bursting and axonal spike propagation, available physiological knowledge proved *sufficient* to explain the fundamental aspects of PC electroresponsiveness. Several model predictions could be validated against data not used to construct it. Of relevance is that ionic current patterns during simple spikes and complex bursting reflected those reported experimentally. Both Na and Ca current transfer between AIS and dendrites allowed to sustain spontaneous firing and complex bursting, as anticipated by previous Ca- and Na-hypotheses. Moreover, while the model was calibrated against the f/I relationship generated by current steps, it also correctly reproduced the f/I relationship generated by ramp current injection in the soma (Roth and Hausser, 2001). Consistent with experimental findings, major firing pattern alterations could be observed when axonal or dendritic compartments were severed or with gene KO of critical ionic channels. Three main model predictions will require experimental testing. First, bistability may occur only through Ca and Na channel down-regulation. Secondly, RNs may become secondary spike generators when the AIS is inactivated at high-frequency and depolarizing currents propagate from the dendrites. Thirdly, K channels with A-type properties may be expressed in the RNs in order to effectively limit spike transmission frequency. As a whole, the picture emerges of a complex neuron with multiple mechanisms of spike generation, in which firing patterns transmitted to the DCN faithfully reproduce those observed in the soma only at <200 Hz but not at higher frequencies. While some predictions await for experimental testing, the PC model may be used to investigate the impact of ionic channels and synaptic input patterns and to investigate *in computo* the internal dynamics of the cerebellar network (Solinas et al., 2010) in physiological and pathological states.

### Conflict of interest statement

The authors declare that the research was conducted in the absence of any commercial or financial relationships that could be construed as a potential conflict of interest.
